# Preclinical evaluation of AT-527, a novel guanosine nucleotide prodrug with potent, pan-genotypic activity against hepatitis C virus

**DOI:** 10.1371/journal.pone.0227104

**Published:** 2020-01-08

**Authors:** Steven S. Good, Adel Moussa, Xiao-Jian Zhou, Keith Pietropaolo, Jean-Pierre Sommadossi

**Affiliations:** Atea Pharmaceuticals, Inc., Boston, Massachusetts, United States of America; Consejo Superior de Investigaciones Cientificas, SPAIN

## Abstract

Despite the availability of highly effective direct-acting antiviral (DAA) regimens for the treatment of hepatitis C virus (HCV) infections, sustained viral response (SVR) rates remain suboptimal for difficult-to-treat patient populations such as those with HCV genotype 3, cirrhosis or prior treatment experience, warranting development of more potent HCV replication antivirals. AT-527 is the hemi-sulfate salt of AT-511, a novel phosphoramidate prodrug of 2’-fluoro-2’-C-methylguanosine-5'-monophosphate that has potent *in vitro* activity against HCV. The EC_50_ of AT-511, determined using HCV laboratory strains and clinical isolates with genotypes 1–5, ranged from 5–28 nM. The active 5'-triphosphate metabolite, AT-9010, specifically inhibited the HCV RNA-dependent RNA polymerase. AT-511 did not inhibit the replication of other selected RNA or DNA viruses *in vitro*. AT-511 was approximately 10-fold more active than sofosbuvir (SOF) against a panel of laboratory strains and clinical isolates of HCV genotypes 1–5 and remained fully active against S282T resistance-associated variants, with up to 58-fold more potency than SOF. *In vitro*, AT-511 did not inhibit human DNA polymerases or elicit cytotoxicity or mitochondrial toxicity at concentrations up to 100 μM. Unlike the other potent guanosine analogs PSI-938 and PSI-661, no mutagenic O^6^-alkylguanine bases were formed when incubated with cytochrome P450 (CYP) 3A4, and AT-511 had IC_50_ values ≥25 μM against a panel of CYP enzymes. In hepatocytes from multiple species, the active triphosphate was the predominant metabolite produced from the prodrug, with a half-life of 10 h in human hepatocytes. When given orally to rats and monkeys, AT-527 preferentially delivered high levels of AT-9010 in the liver *in vivo*. These favorable preclinical attributes support the ongoing clinical development of AT-527 and suggest that, when used in combination with an HCV DAA from a different class, AT-527 may increase SVR rates, especially for difficult-to-treat patient populations, and could potentially shorten treatment duration for all patients.

## Introduction

There are approximately 71 million people globally who are chronically infected with hepatitis C virus (HCV). A significant number of those with chronic infection will develop cirrhosis, hepatocellular carcinoma or liver failure, resulting in approximately 400,000 deaths each year [1]. Recently approved combination regimens of DAAs, such as sofosbuvir (SOF) plus ledipasvir or velpatasvir and glecaprevir plus pibrentasvir, have not only drastically improved efficacy outcomes, but have decreased treatment side effects compared to the interferon-containing regimens of the past [2]. Indeed, these regimens have produced sustained virologic response (SVR) rates greater than 90%, and with treatment duration of 8–12 weeks, depending on the regimen and patient population, have resulted in sustained virologic cures. Despite these recent advances, significant challenges remain in managing difficult-to-treat populations including those with HCV genotype (GT) 3 infection, cirrhosis or prior treatment experience. These populations typically require at least 12 weeks of treatment, often in combination with ribavirin, which increases the risk of serious side effects [3], or they are not able to receive treatment regimens containing a protease inhibitor due to their advanced liver disease [4]. Therefore, an ideal regimen would have high efficacy based on shorter treatment duration for most, if not all, patient populations, with an improved safety profile that includes less drug-drug interaction potential compared to some currently approved regimens.

**Fig 1:**
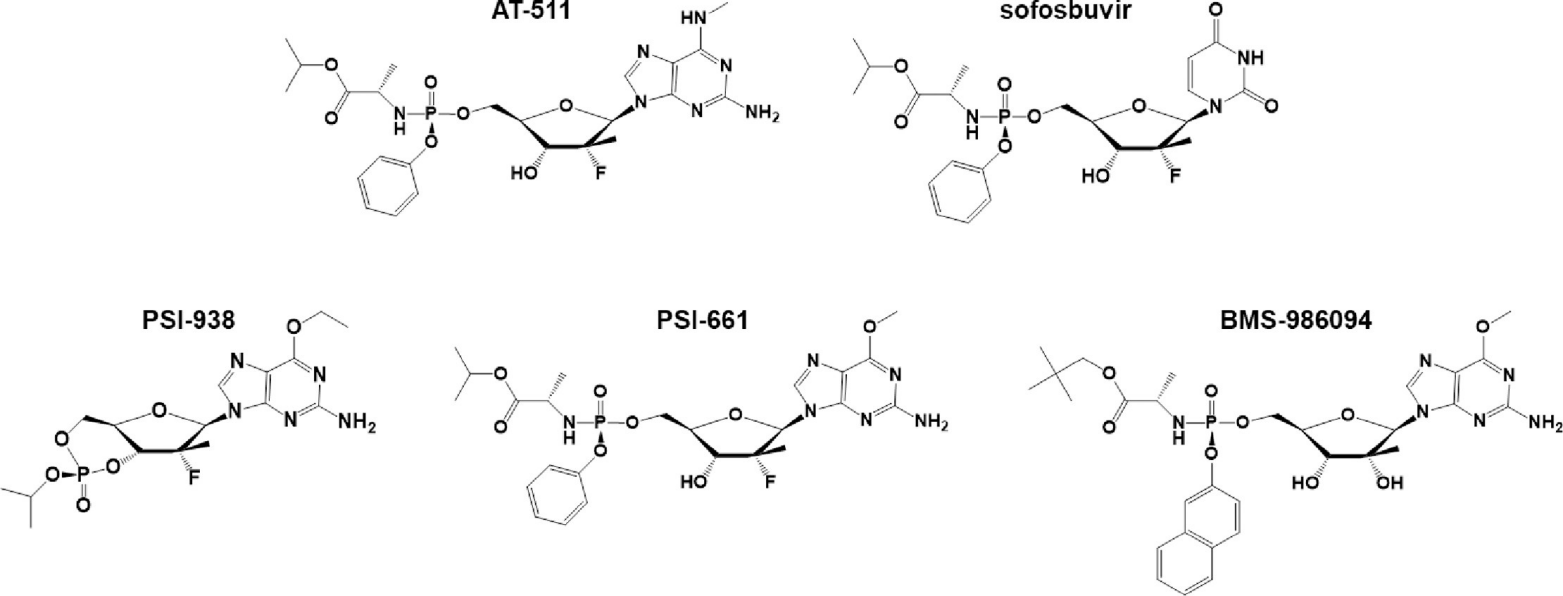
Chemical structures of AT-511, sofosbuvir (SOF), PSI-938, PSI-661 and BMS-986094.

For the treatment of HCV, DAAs have been developed that target different critical steps in the HCV life cycle. One such target of interest is the RNA-dependent RNA polymerase (RdRp), encoded by the NS5B region of the HCV genome, which is essential for viral replication. Thus, nucleotide analogs that can be converted to their triphosphate analog within cells, incorporated into nascent HCV RNA, and block viral replication are preferred candidates since they have proven to be potent, pan-genotypic inhibitors with a high barrier to drug resistance. In addition, the apparent improved antiviral activity of guanosine analogs, compared to pyrimidine analogs such as SOF [5], had prompted a search for modified guanosine analogs containing a proven substituted ribose moiety with a monophosphate prodrug. Previously identified guanosine analogs such as PSI-938 (a cyclic phosphate prodrug of 2’-fluoro-2’-C-methylguanosine-5’-monophosphate) and BMS-986094 (a prodrug of 2’-C-methylguanosine-5’-monophosphate) had excellent activity against HCV replication, but their development was halted due to observed hepatic and cardiac toxicity, respectively [6, 7]. The structures of AT-527, SOF, PSI-938 and BMS-986094 as well as PSI-661, a structurally-related HCV inhibitor that showed potent *in vitro* activity against HCV [8], are presented in **Fig 1**.

**Fig 2:**
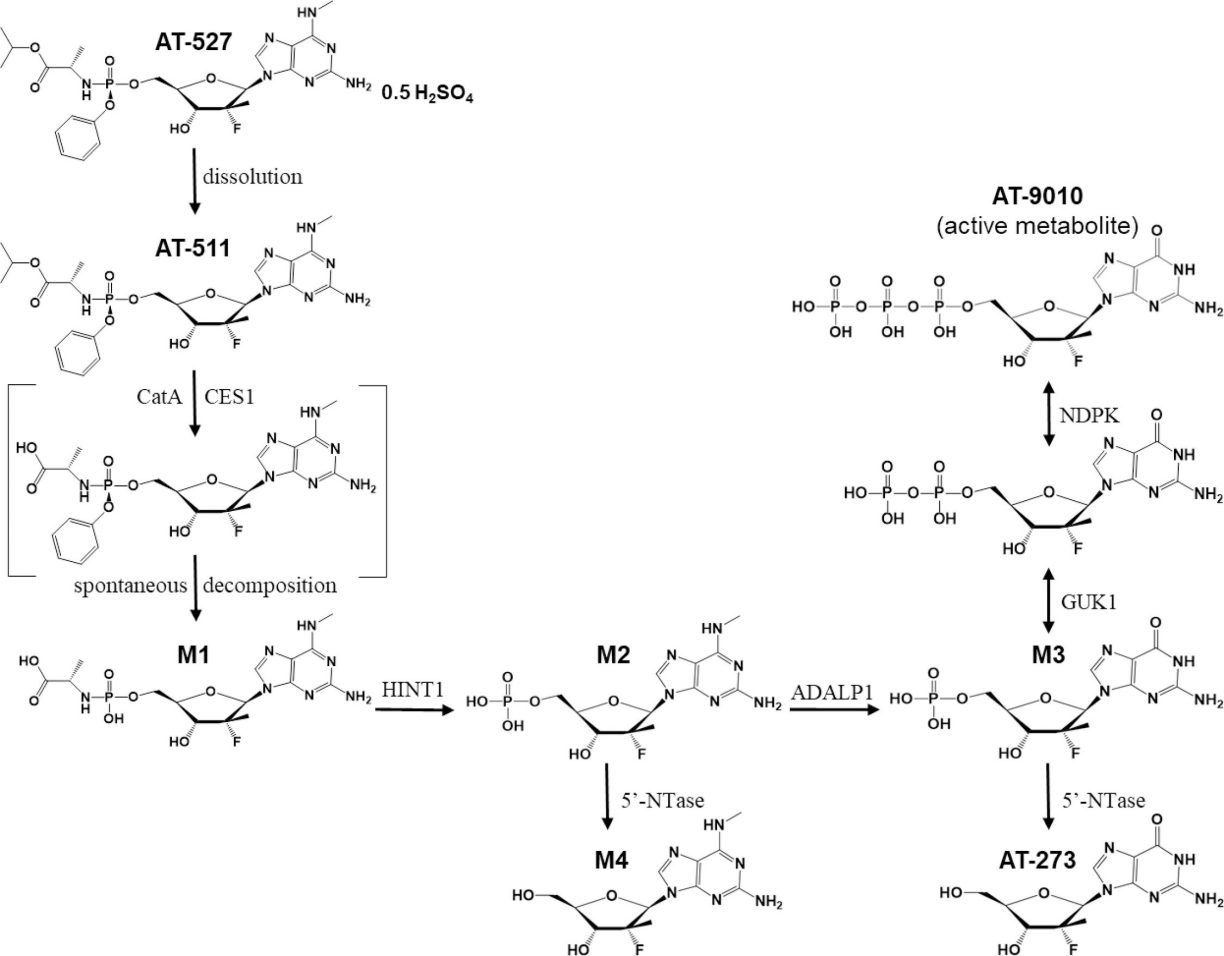
Putative metabolic pathway for AT-527. When dissolved, AT-527 releases its free base AT-511. Sequential hydrolysis, catalyzed by human cathepsin A (CatA) and/or carboxylesterase 1 (CES1) followed by spontaneous cleavage of the then unstable phenolic moiety, produces the L-alanyl intermediate (M1). Removal of the amino acid moiety by histidine triad nucleotide-binding protein 1 (HINT1) results in metabolite M2 which can then be converted to M3 by adenosine deaminase like protein 1 (ADALP1). M3 is further anabolized sequentially by guanylate kinase 1 (GUK1) and nucleoside diphosphate kinase (NDPK) to the pharmacologically active triphosphate, AT-9010. Both M2 and M3 can be dephosphorylated by 5’-nucleotidase (5’-NTase) to their respective nucleosides M4 and AT-273.

We are presently developing AT-527, whose putative metabolic pathway is presented in **Fig 2**. AT-527 is the hemi-sulfate salt of AT-511, a phosphoramidate prodrug of 2’-fluoro-2’-C-methylguanosine-5’-monophosphate. The phosphoramidate moiety of AT-527 is identical to that of SOF [9] and is presumed to be subject to the same initial metabolic activation pathway leading to the unique nucleotide monophosphate (MP) still containing the N^6^-methyl modification (M2), that is, hydrolysis catalyzed by human cathepsin A (CatA) and/or carboxylesterase 1 (CES1) producing the L-alanyl intermediate (M1), followed by removal of the amino acid moiety by histidine triad nucleotide-binding protein 1 (HINT1) resulting in the MP metabolite, M2. As with PSI-938, M2 is likely converted to M3 by adenosine deaminase like protein 1 (ADALP1; [10]) and further anabolized sequentially by guanylate kinase 1 (GUK1) and nucleoside diphosphate kinase (NDPK) to the pharmacologically active triphosphate (TP), AT-9010. Presumably, both M2 and M3 can be dephosphorylated by 5’-nucleotidase (5’-NTase) to their respective nucleosides M4 and AT-273 (**Fig 2**). Thus, AT-527 possesses a unique structural feature with an N^6^-methyl group which critically differentiates it from other guanosine nucleotide analogs with respect to drug metabolism and provides the compound with a favorable preclinical safety profile while maintaining highly potent antiviral activity, supporting its clinical development.

## Results

### *In vitro* antiviral activity of AT-511

AT-511, the free base of AT-527, was used for most of the *in vitro* experiments to measure the effective concentration at which virus replication is inhibited by 50% (EC_50_) and 95% (EC_95_), and the concentration required to produce a toxic effect on 50% of the exposed cells (TC_50_). The salt form of AT-511, AT-527, was used in the animal studies because of its increased aqueous solubility, thus enhancing oral absorption.

**Fig 3:**
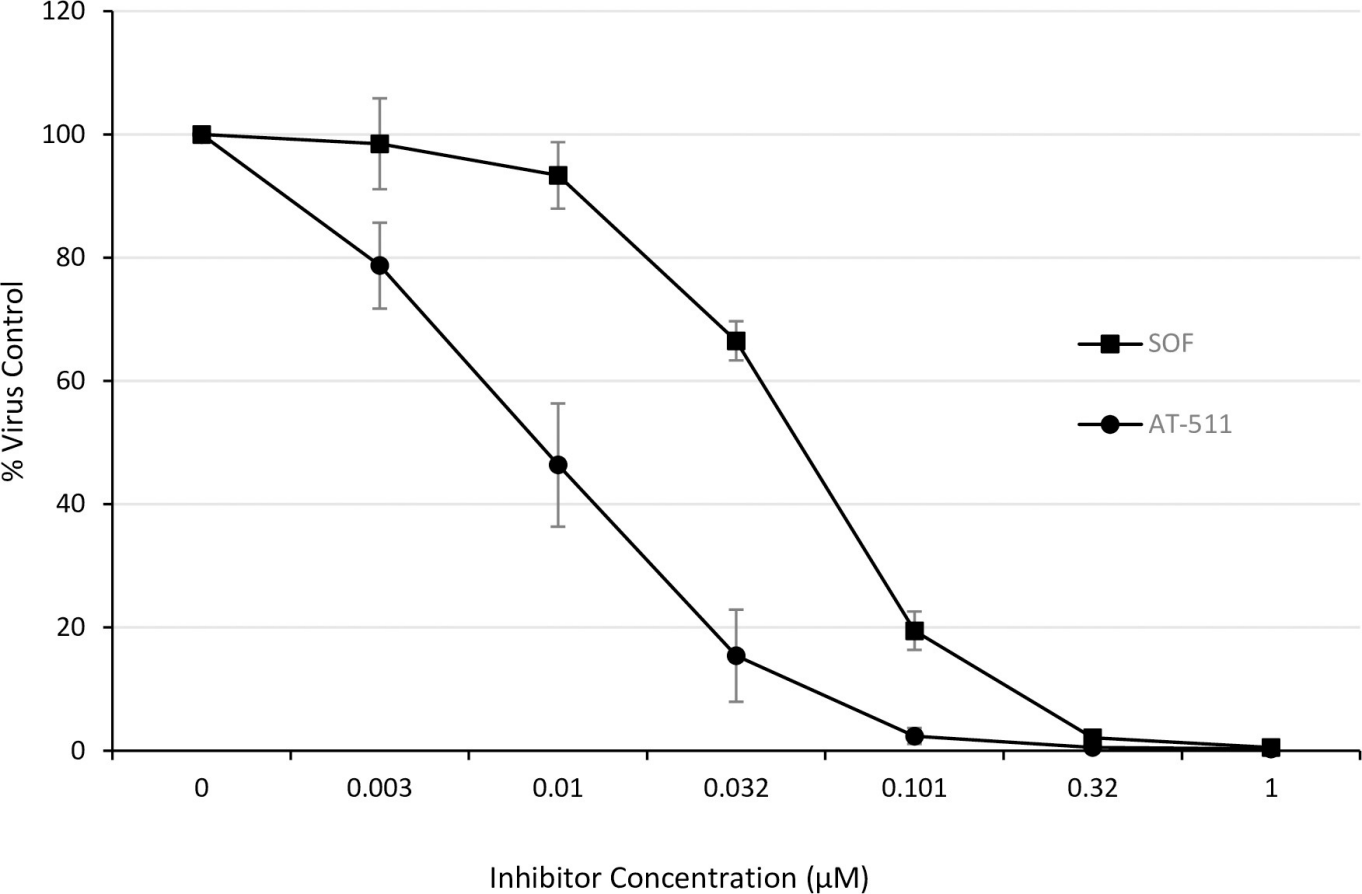
Inhibition of viral replication in HCV GT1b replicons treated with AT-511 or SOF. Huh-luc/neo-ET cells stably transfected with the HCV GT1b NS3-NS5B coding sequence were incubated with serial dilutions of AT-511 or sofosbuvir (SOF) in parallel and anti-HCV activity was measured as described in Methods. Data are expressed as mean ± SE, n = 8 assays in triplicate.

In initial experiments, an HCV GT1b replicon system in stably transfected Huh-7 cells was used to evaluate the anti-HCV activity and the cytotoxicity of AT-511 using the Britelite and XTT reagents, respectively. SOF, which was used as a positive control, had a mean EC_50_ value of 48 ± 10 nM (range 33 to 68 nM) and a mean EC_95_ value of 270 ± 70 nM (range 197 to 387 nM). Six different blinded batches of AT-511 (n = 9 determinations) gave a mean EC_50_ value of 5 ± 3 nM (range 0.8 to 12 nM) and a mean EC_95_ value of 25 ± 13 nM (range 3 to 50 nM), demonstrating that AT-511 is roughly 10-fold more potent than SOF (**Fig 3; S1 Table**). Neither AT-511 nor SOF showed toxicity to the replicon cells up to the highest concentrations tested (100 μM for AT-511; 1 μM for SOF).

The antiviral activities of AT-511 and SOF were also determined using Huh-7 replicon cells stably-transfected with the NS5B region of HCV GT1b (HCV genotype 1b, Con-1) in the PhenoSense HCV NS5B assay. Concentrations of SOF needed to achieve EC_50_ and EC_95_ levels in this laboratory strain (89 and 545 nM, respectively) were up to 9 times greater than those for AT-511 (13 and 61 nM, respectively) (**Table 1**). An additional five Huh-7 cell lines containing the NS5B regions of various HCV genotypes were tested with AT-511 and SOF (**Table 1**). Similar to the reference virus, the EC_50_ value was, on average, 9-fold higher for SOF (11-fold higher for EC_95_) as compared to AT-511, with potency ratios ranging from 5.5 to 11.4 for the different strains. Additionally, the range in EC_50_ values for SOF (51–287 nM) was 5.6-fold across the genotypes tested, compared with a tighter 3.1-fold range (9–29 nM) for AT-511.

**Table 1:** Pan-genotypic antiviral activity of AT-511 in a panel of laboratory HCV replicons.

HCV Genotype	Reference Strain	GenBank Accession ID	EC_50_ (nM)			EC_95_ (nM)		
			AT-511	SOF	SOF/AT-511	AT-511	SOF	SOF/AT-511
1a	H77	NC_004102	12.8	83.4	6.5	64.2	719	11.2
1b	Con-1*	AJ238799	12.5	89.4	7.2	61.0	545	8.9
2a	JFH-1	AB047639	9.2	50.7	5.5	ND	ND	ND
3a	S52	GU814263	10.3	117	11.4	50.0	700	14
4a	ED43	GU814265	14.7	158	10.7	ND	ND	ND
5a	SA13	AF064490	28.5	287	10.1	ND	ND	ND

Antiviral activity was measured using the PhenoSense assay as described in the Methods.

Cell viability was measured using the alamarBlue^®^ assay as described in the Methods; neither test article affected

viability up to the highest concentration tested (10,000 nM for AT-511 and 100,000 nM for SOF).

*Used as Control Reference for all PhenoSense assays

EC_50_, effective concentration required to inhibit virus replication by 50%

EC_95_, effective concentration required to inhibit virus replication by 95%

ND, not determined

In addition, EC_50_ values across eight different Huh-7 replicons transiently-transfected with the NS5B regions of HCV derived from clinical isolates of various genotypes were, on average, 8-fold higher for SOF than AT-511, with potency ratios ranging from 4 to 12 (**Table 2**). The range in SOF EC_50_ values (23–126 nM) was 5.5-fold, whereas that for AT-511 covered only a 2.3-fold range (6–14 nM). The EC_95_ potency ratios (4 to 14) and variabilities (195–1020 nM and 5.2-fold for SOF; 33–80 nM and 2.5-fold for AT-511) were similar across the patient-derived genotypes as well (**Table 2**). These results demonstrate that AT-511 exhibits pan-genotypic anti-HCV activity which is consistently more potent than SOF against all HCV genotypes tested.

**Table 2:** Pan-genotypic antiviral activity of AT-511 in a panel of replicons containing HCV constructs from clinical isolates.

Genotype	EC_50_ (nM)			EC_95_ (nM)		
	AT-511	SOF	SOF/AT-511	AT-511	SOF	SOF/AT511
1a	10.5	62.7	6.0	60.8	508	8.4
1b	10.6	86.0	8.1	72.3	642	8.9
2a	6.2	22.5	3.7	45.4	195	4.3
2b	6.3	44.8	7.0	32.5	295	9.1
3a-1	11.8	126	10.7	59.3	690	11.6
3a-2	10.4	123	11.8	56.5	808	14.3
4a	9.9	74.9	7.6	74.4	681	9.2
4d	14.0	93.7	6.7	79.9	1020	12.8

Antiviral activity was measured by the PhenoSense assay as described in the Methods.

Cell viability was measured using the alamarBlue® assay as described in the Methods; neither test article affected viability up to the highest concentration tested (10,000 nM for AT-511 and 100,000 nM for SOF).

EC_50_, effective concentration required to inhibit virus replication by 50%

EC_95_, effective concentration required to inhibit virus replication by 95%

When several HCV laboratory strains and clinical isolates containing the C316N, L159F or S282T resistance-associated variants (RAVs) were tested using the PhenoSense assay, the EC_50_ and EC_95_ values were, respectively, 8- and 10-fold higher for SOF compared to AT-511, *except* for the three strains containing the S282T genetic variation **(Table 3).** For those with this S282T polymorphism, SOF was significantly less effective in inhibiting viral replication compared to its response using the reference strain lacking the S282T variant, requiring 15- and 19-fold more drug to achieve EC_50_ and EC_95_ levels, respectively. On the other hand, the shift in EC_50_ and EC_95_ values for AT-511 were only 2.5- and 4.2-fold respectively. Therefore, AT-511 showed little evidence of cross-resistance in any of the HCV strains tested and was significantly more potent (>40-fold) than SOF against RAVs containing the S282T variant.

**Table 3:** Inhibition by AT-511 and SOF of HCV strains containing resistance-associated variants (RAVs).

HCV Genotype	Reference Strain with Genetic Variation	EC_50_ (nM)			EC_95_ (nM)		
		AT-511	SOF	SOF/AT-511	AT-511	SOF	SOF/AT-511
1b	Con-1_L159F	17.3	111	6.4	107	715	6.9
1b	Con-1_S282T	17.9	950	53.1	124	7230	58.3
1a	H77_C316N	9.6	57.2	6.0	55.2	469	8.5
1a	H77_S282T	22.0	1397	64	179	7230	40.4
2b	2_L159F	10.2	79.2	7.8	49.5	539	10.9
1a	2c1_L159F	13.0	115	8.8	95.3	1068	11.2
1a	2c2_L159F_S282T	38.7	1619	41.8	313	16950	54.2
1a	2_C316N	7.7	73.9	9.6	42.7	473	11.1

Antiviral activity was measured by the PhenoSense assay as described in the Methods.

Cell viability was measured using the alamarBlue® assay as described in the Methods; neither test article affected viability up to the highest concentration tested (10,000 nM for AT-511 and 100,000 nM for SOF).

EC_50_, effective concentration required to inhibit virus replication by 50%

EC_95_, effective concentration required to inhibit virus replication by 95%

To test the specificity of AT-511, a panel of selected RNA and DNA viruses was incubated with the drug at concentrations up to 100 μM, and the EC_50_ and TC_50_ values compared to the response from control compounds effective against these viruses (**Table 4**). Whereas nanomolar levels of AT-511 strongly inhibited HCV, much higher concentrations resulted in only weak activity against zika virus and human rhinovirus (HRV). At 100 μM, AT-511 was not active against 8 other RNA and DNA viruses (**Table 4**).

**Table 4:** Inhibition of various RNA and DNA viruses by AT-511 compared to control compounds.

Virus	Cells	Control Compound	Control Compound		AT-511	
			EC_50_ (μM)	TC_50_ (μM)	EC_50_ (μM)	TC_50_ (μM)
HIV-1	CEM-SS	Zidovudine	0.004	>0.50	>100	>100
HSV-1	Vero	Acyclovir	1.76	>100	>100	>100
Flu A	MDCK	Oseltamivir	0.16	>10.0	>100	>100
Flu B	MDCK	Oseltamivir	0.42	>10.0	>100	>100
Zika	Vero	Ribavirin	5.65	38.8	8.07	>100
HRV	H1-HeLa	Ribavirin	17.1	40.6	18.6	>100
AdV	HeLa	Ribavirin	0.76	11.7	>100	>100
CoV	MRC-5	Ribavirin	3.85	>100	>100	>100
RSV	HEp2	TMC-353121	0.003	>0.10	>100	>100
HBV	AD38	TDF	0.23	>1.0	>100	>100

Antiviral activity and cell viability were measured as described in the Methods.

EC_50_, effective concentration required to inhibit virus replication by 50%

TC_50_, threshold concentration required to decrease cell viability by 50%

HIV-1, human immunodeficiency virus type 1; HSV-1, herpes simplex virus type 1; Flu A and B, influenza virus types A and B; HRV, human rhinovirus; AdV, adenovirus; CoV, coronavirus; RSV, respiratory syncytial virus; HBV, hepatitis B virus; TDF, tenofovir disoproxil fumarate

### *In vitro* selectivity

AT-9010 was a potent and specific inhibitor of the HCV RdRp *in vitro* with a 50% inhibitory concentration (IC_50_) of 0.15 μM (mean of two assays) when tested as an alternate substrate for GTP, whereas it only weakly inhibited that polymerase (IC_50_ 40.3 μM; single assay) as a competitive inhibitor of ATP. Moreover, AT-9010 was inactive (IC_50_ >100 μM) when tested as an inhibitor of human DNA polymerases α, β and γ, as compared to the IC_50_ values observed for the respective positive controls aphidicolin (2.63 μM), lithocholic acid 2.91 (μM) and 2’-fluoro-2’-dGTP (10.4 μM).

Since the toxicity of several nucleoside triphosphate analogs are thought to be the result of their ability to inhibit human mitochondrial RNA polymerase (POLRMT), thus disrupting mitochondrial RNA transcription and protein synthesis [11], the potential for AT-9010 to inhibit POLRMT was assessed in parallel with the active TP of SOF (2’-fluoro-2’-C-methyl UTP), and the active TP of BMS-986094 (2’-C-methyl GTP), a drug known to cause toxicity via its inhibition of POLRMT [12]. Although the active form of all three drugs were utilized by POLRMT *in vitro*, and this incorporation prevented subsequent cycles of nucleotide addition (**S1**–**S3 Figs**), their efficiency of incorporation (*k*_*pol*_*/K*_*d*,*app*_) was poor compared to the corresponding natural nucleotide (**Table 5**). The incorporation efficiencies of AT-9010 and SOF TP were within two-fold, whereas the TP of BMS-986094 was incorporated by POLRMT 55 times more efficiently than SOF TP.

**Table 5:** Kinetic parameters for POLRMT-catalyzed nucleotide incorporation.

Nucleotide	k_pol_ (s^-1^)	K_d,app_ (μM)	k_pol_/K_d, app_ (μM^-1^s^-1^)	Relative efficiency*
UTP^a^	17 ± 1	29 ± 2	0.60 ± 0.05	-
SOF TP	34 ± 5 x 10^−5^	590 ± 250	5.8 ± 2.6 x 10^−7^	1.0 x 10^−6^
GTP^a^	21 ± 1	5.5 ± 0.5	3.8 ± 0.4	-
BMS-986094 TP	51 ± 2 x 10^−3^	240 ± 26	2.1 ± 0.2 x 10^−4^	5.5 x 10^−5^
AT-9010	17 ± 2x 10^−4^	204 ± 94	8.3 ± 4.0 x 10^−6^	2.2 x 10^−6^

*Relative efficiency is defined as: (k_pol_/K_d,app_)_incorrect_/(k_pol_/K_d,app_)_correct_.

^a^Kinetic parameters for the nucleotide controls were taken from [11].

To determine whether the minimal interaction of AT-9010 with POLRMT could result in deleterious effects on mitochondrial function, AT-511 was incubated with K562 cells in cell culture medium with galactose substituted for glucose, thereby assessing the ability of the mitochondrial electron transport system to maintain cellular ATP levels without contributions from glycolysis. As measures of mitochondrial toxicity, membrane integrity and inhibition of cellular ATP levels were assessed and compared to K562 cells incubated in glucose-containing medium. AT-511 at concentrations up to 20 μM was not cytotoxic and did not alter K562 cell membrane integrity or affect mitochondrial function in the presence or absence of galactose (**Table 6**). The positive control compound digitonin induced necrosis and affected mitochondrial function in the presence of glucose as well as in its absence. CCCP, which permeabilizes mitochondrial inner membranes, inhibited cellular ATP levels in K562 cells in the presence of glucose, but more severely in its absence (**Table 6**).

**Table 6:** Cytotoxicity and mitochondrial toxicity in human cell lines.

Compound	K562 cells			PC3 cells		
	Cytotoxicity TC_50_ (μM)	MI (Gal)^a^ IC_50_ (μM)	MF (Gal)^b^ IC_50_ (μM)	Cytotoxicity TC_50_ (μM)	Mitogenesis IC_50_ (μM)	
					SDH-A	COX-1
Digitonin	8.1	31.9	27.9	27.2	40.7	32.6
CCCP*	8.8	7.0	<0.6	17.7	13.5	15.1
Chloramphenicol*	50.9	>100	>100	>100	>100	95.3
ddC*	>20	>20	>20	>20	>20	1.1
AT-511	>20	>20	>20	>20	>20	>20

^a^Cell membrane integrity in glucose-free, galactose containing medium after 2 h incubation, as a measure of cell necrosis

^b^Mitochondrial function in glucose-free, galactose containing medium after 2 h incubation, as measured by inhibition of cellular ATP levels

*Compounds used as positive effector controls

In further attempts to assess mitochondrial toxicity, the effects of AT-511 on mitochondrial biogenesis in PC3 cells were assessed by measuring levels of the nuclear-DNA-encoded SDH-A protein and the mitochondrial-DNA-encoded COX-1 protein. After 5 days of treatment at concentrations up to 20 μM, AT-511 was not cytotoxic and had no effect on expression levels of either protein (**Table 6**). Digitonin and CCCP were generally cytotoxic to PC3 cells, whereas chloramphenicol, which arrests mitochondrial protein synthesis, and ddC, which inhibits mitochondrial DNA replication, selectively reduced COX-1 levels (**Table 6**).

In addition to the cell lines listed in **Tables 4** and **6**, AT-511 showed no cytotoxicity at the highest concentrations tested (up to 100 μM) in any of the antiviral replicon assays or when incubated with mouse, rat, dog, monkey or human hepatocytes. The cytotoxicity of AT-511 was further evaluated in human induced pluripotent stem cell (iPS) cardiomyocytes and in granulocyte macrophage (GM) and erythroid (E) human bone marrow progenitor cells. AT-511 showed no toxicity in the cardiomyocyte or bone marrow stem cell assays (TC_50_ > 100 μM), whereas the positive control compounds had the expected cytotoxic effects (TC_50_ values of 18 μM for doxazosin with cardiomyocytes and 1 and 2 μM for AZT in the GM and E cell assays, respectively).

**Fig 4:**
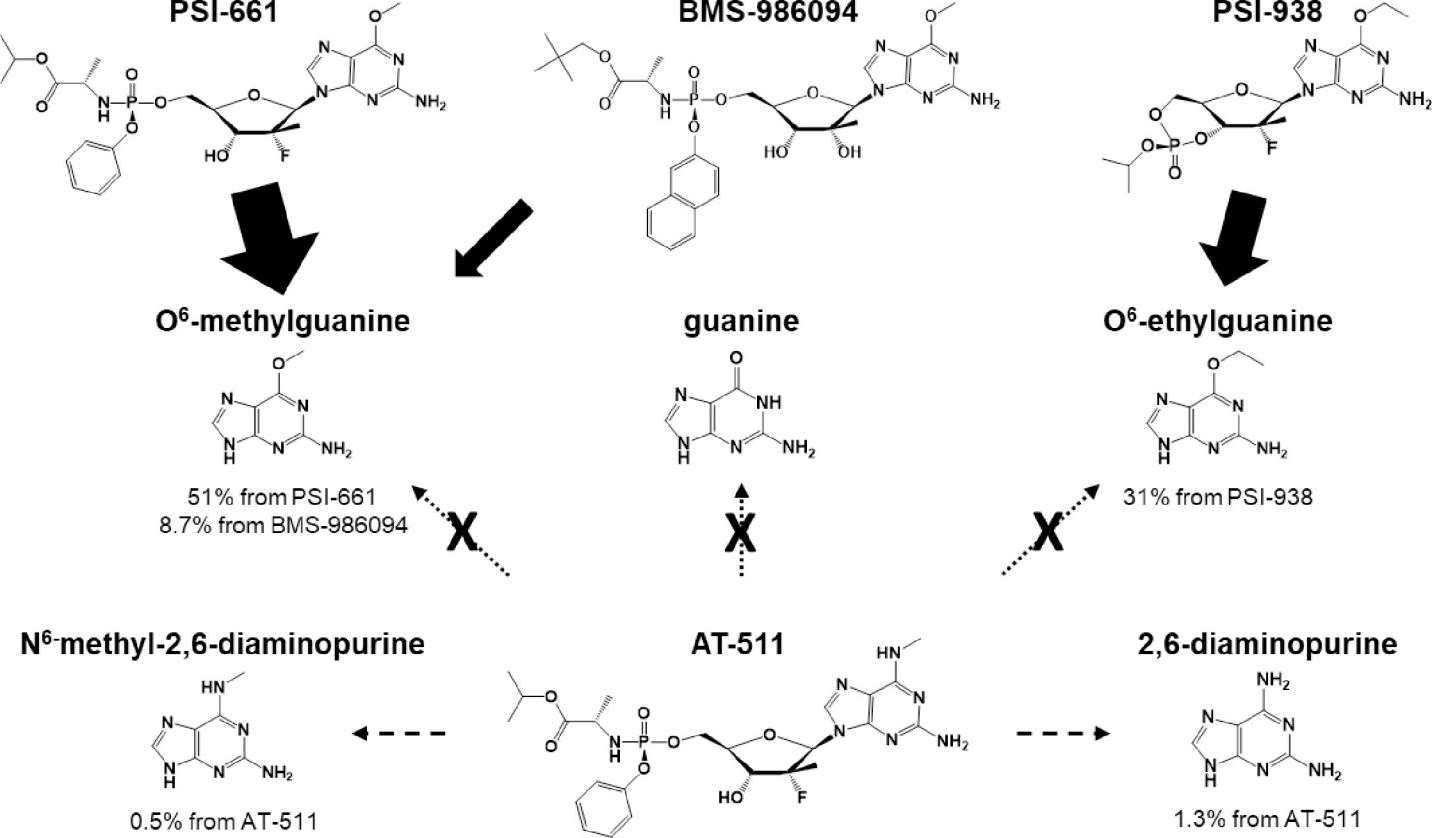
CYP3A4-mediated metabolism of PSI-661, BMS-986094, PSI-938 and AT-511. Values indicate the extent of the 5 μM starting concentration of each antiviral that was converted to the indicated nucleobase after a 2-h incubation with rhCYP3A4.

Other prodrugs of M3, such as PSI-661 and PSI-938, have been discontinued or have failed due to toxicity in the clinic likely due to the formation of mutagenic O^6^-alkylated nucleobase metabolites [13–15]. The formation of these metabolites was assessed in separate 2-h incubations of AT-511, PSI-661, PSI-938 and BMS-986094 at 5 μM initial concentrations with recombinant human cytochrome P450 3A4 (rhCYP3A4) in the presence of NADPH. AT-511 formed low levels of 2,6-diaminopurine and *N*^6^-methyl-2,6-diaminopurine (0.065 ± 0.010 μM and 0.024 ± 0.006 μM; mean ± SD) (**S2 Table**). Guanine, *O*^6^-methylguanine and *O*^6^-ethylguanine were not detected in the incubation medium containing AT-511, as would be expected from its unique structure (**Fig 4**). PSI-938 formed 1.57 ± 0.011 μM of *O*^6^-ethylguanine while PSI-661 and BMS-986094 formed *O*^6^-methylguanine, 2.56 ± 0.057 and 0.434 ± 0.007 μM, respectively. These results indicate that, of the starting drug concentrations, 51% of PSI-661, 31% of PSI-938 and 8.7% of BMS-986094 were converted to known mutagenic O^6^-alkylguanine bases. No such nucleobases were formed from AT-511, and a total of only 1.8% was converted to other modified nucleobases.

To determine if AT-511 inhibits cytochrome P450 (CYP) enzymes, a panel of substrates selective for seven CYP isozymes were separately added to liver microsomes and incubated with the HCV inhibitor. AT-511 had IC_50_ values >30 μM (the highest concentration tested) for CYP1A2, CYP2B6, CYP2C8, CYP2C9, CYP2C19 and CYP2D6, and only weakly inhibited CYP3A4 (25 μM IC_50_) compared to the inhibitors used as positive controls for the enzymes, whose IC_50_ values ranged from 0.01 to 4.6 μM (**S3 Table**). Therefore, AT-511 has low potential for drug-drug interactions mediated by CYP enzymes.

### *In vitro* metabolism

**Fig 5:**
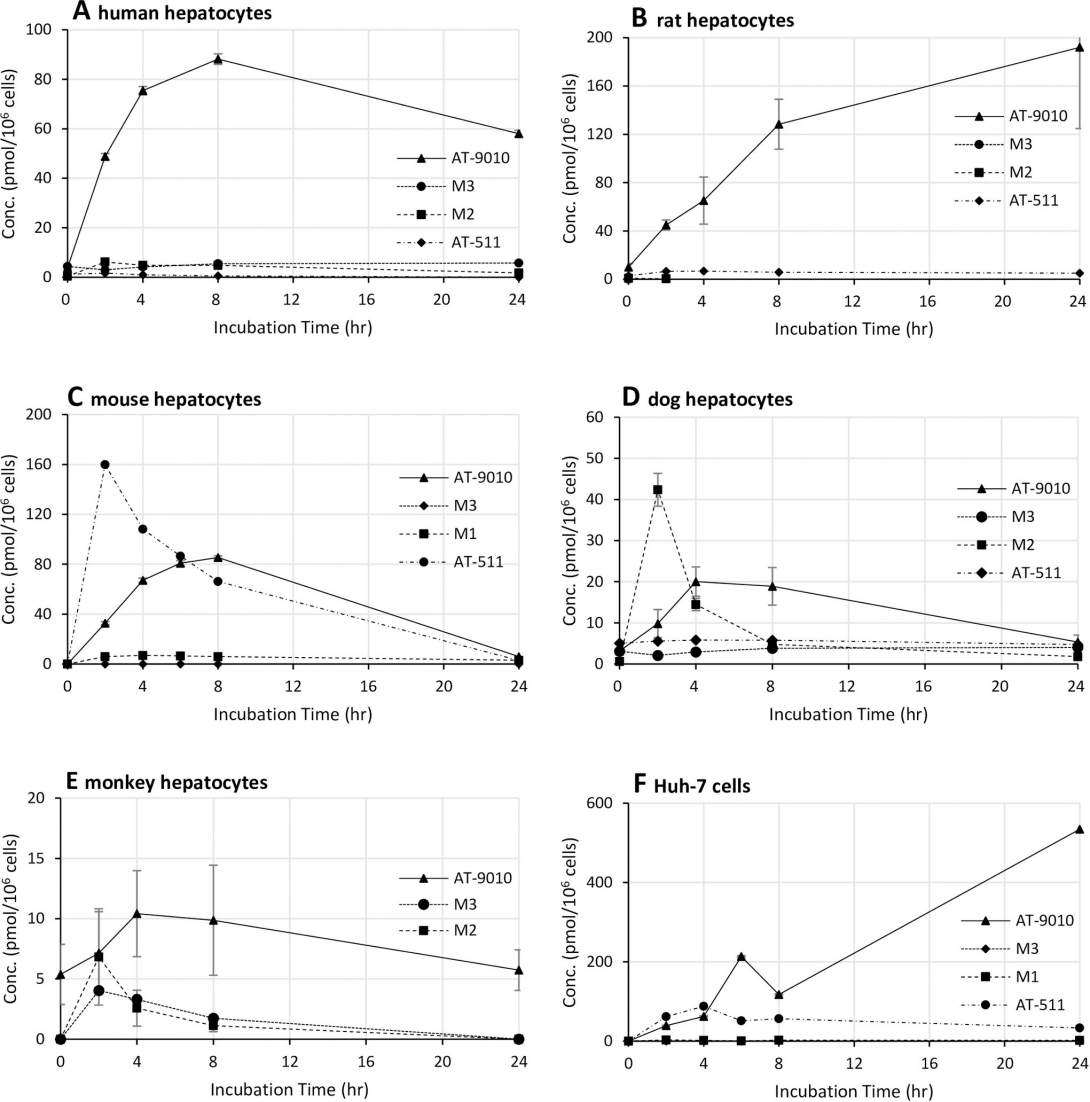
Intracellular concentrations of AT-511 and its phosphorylated metabolites in liver cells. (A) mixed gender human hepatocytes, (B) male rat hepatocytes, (C) male mouse hepatocytes, (D) male dog hepatocytes, (E) male cynomolgus monkey hepatocytes and (F) Huh-7 cells. Cells were incubated for 24 h with 10 μM AT-511 in triplicate, collected, lysed and the lysates measured for concentrations of AT-511, AT-9010, M2 and M3 by LC-MS/MS. Data are expressed as mean ± SD.

The metabolism of AT-511 was characterized in Huh-7 cells (the human hepatocarcinoma cell line used to construct HCV replicons for measuring antiviral activity) and in mouse, rat, dog, monkey and human hepatocytes. In the absence of cells, AT-511 was stable when incubated in culture medium at 37°C for up to 24 h. AT-9010 was the predominant intracellular metabolite measured in all hepatocyte species and Huh-7 cell incubations with 10 μM AT-511 (**Fig 5**) and, except in mouse hepatocytes, far exceeded intracellular AT-511 concentrations, demonstrating that the active TP was efficiently formed from its phosphoramidate protide in liver cells. In hepatocyte incubations, the relative levels of AT-9010 formed, and its accumulation over 24 h (expressed as area under the curve or AUC_0-24h_; **Table 7**), from most to least abundant, were rat>human >mouse >dog/monkey.

**Table 7:** Intracellular levels of AT-9010 in hepatic cells.

Cell Type	C_max_ (pmol/10^6^ cells)^a^	AUC_0-24_ (pmol*h/10^6^ cells)^b^
Huh-7 cells	420.3 ± 161.4	5051
Rat hepatocytes	192.0 ± 67.3	3114
Mouse hepatocytes	85.4 ± 0.4	1177
Human hepatocytes	69.2 ± 8.8	1363
Dog hepatocytes	15.4 ± 6.6	229
Monkey hepatocytes	10.4 ± 0.2	195

Suspended Huh-7 cells or plated hepatocytes were incubated with 10 μM AT-511 for 0, 2, 4, 6, 8 and 24 h, washed, lysed and the active TP measured by LC-MS/MS. Data are expressed as mean ± SD.

^a^C_max_ = Maximum concentration across the time points measured

^b^AUC_0-24_ = Area under the curve for 0–24 h; calculated using the trapezoidal rule.

The 5’-TP of M2 (**Fig 2**), still containing the N^6^-methyl moiety in the purine ring, was not detected. In fact, there was no other quantifiable TP found in any of the hepatocytes at any time point. M3 was the predominant MP metabolite observed in Huh-7 cells and all hepatocytes except for mouse. A lower amount of the MP, M2, was detected, consistent with the hypothesis that the phosphoramidate is metabolized to M2 which, in turn, is efficiently oxidatively deaminated to M3 prior to stepwise phosphorylation to the active TP, AT-9010. Low to undetectable intracellular levels (<10 pmol/10^6^ cells) of the nucleoside metabolites, AT-273 and M4, presumably formed through dephosphorylation, were observed in mouse, dog and human hepatocytes (not measured in monkey hepatocytes). In human hepatocytes incubated with 100 μM AT-511 for 6 h (the time point when the maximum concentration of AT-9010 was observed), followed by a washout period of 30 h, the half-life of AT-9010 was 10 h, supporting the potential for once daily dosing in patients.

### Pharmacokinetics

Individual plasma concentrations of AT-511, M1, M4 and AT-273 after a single oral administration of AT-527, the hemi-sulfate salt of AT-511, to rats (500 mg/kg) and monkeys (30, 100 or 300 mg/kg) (**S4**–**S11 Tables**), were used to calculate the mean pharmacokinetic parameters summarized in **Table 8**. The phosphorylated metabolites formed and retained intracellularly due to their negative charges, including the MPs (M2 and M3), the active TP (AT-9010), and the intermediate diphosphate (DP; **Fig 2**), were not measured in plasma samples but levels of these phosphorylated metabolites were quantitated in samples of liver and heart tissue obtained 4 h after each AT-527 dose in rats and monkeys.

**Table 8:** Rat and monkey pharmacokinetic parameters following administration of a single oral dose of AT-527.

Analyte^a^	Species	Dose (mg/kg)	C_max_^b^ (nmol/mL)	T_max_^c^ (h)	AUC_0-last_^d^ (nmol*h/mL)	MRT_0-last_ ^e^ (h)
AT-511	Rat	500	0.12 ± 0.04	0.29 ± 0.07	0.10 ± 0.04	1.30 ± 0.57
	Monkey	30	0.11 ± 0.06	1.17 ± 0.76	0.30 ± 0.03	17.0 ± 5.0
	Monkey	100	1.35 ± 1.06	1.33 ± 0.58	1.89 ± 1.08	5.28 ± 3.25
	Monkey	300	0.86 ± 0.59	3.33 ± 1.15	2.75 ± 0.71	4.99 ± 2.22
M1	Rat	500	40.8 ± 9.7	0.58 ± 0.14	77.9 ± 20.3	2.54 ± 0.41
	Monkey	30	0.94 ± 0.61	4.67 ± 2.31	5.44 ± 2.76	7.06 ± 1.07
	Monkey	100	4.24 ± 2.84	3.00 ± 1.73	25.5 ± 10.1	7.22 ± 0.48
	Monkey	300	4.44± 4.74	4.67 ± 1.16	35.6 ± 26.4	11.4 ± 3.6
M4	Rat	500	28.8 ± 8.5	3.50 ± 0.87	207.4 ± 56.1	6.41 ± 1.06
	Monkey	30	0.67 ± 0.14	14.7 ± 8.1	18.2 ± 1.7	22.4 ± 2.4
	Monkey	100	1.30 ± 0.49	15.3 ± 7.6	39.2 ± 15.0	22.3 ± 4.1
	Monkey	300	1.66 ± 0.49	17.3 ± 11.5	53.9 ± 31.7	26.6 ± 9.6
AT-273	Rat	500	2.50 ± 0.52	6.34 ± 0.58	40.3 ± 10.9	17.5 ± 2.1
	Monkey	30	0.14 ± 0.01	16.0 ± 6.9	5.42 ± 1.03	26.9 ± 1.9
	Monkey	100	0.44 ± 0.17	4.00 ± 0.0	10.1 ± 2.6	23.7 ± 3.8
	Monkey	300	0.31 ± 0.05	17.3 ± 11.5	12.2 ± 6.1	27.9 ± 8.1

Blood samples were collected up to 72 h post dose, and plasma concentrations of the analytes measured by LC-MS/MS. Data are expressed as mean ± SD of 3 male and 3 female rats (combined) and of 3 male monkeys.

^a^The analytes in the putative metabolic pathway for AT-527 are shown in **Fig 2**.

^b^C_max_ = Maximum concentration across the time points measured

^c^T_max_ = Time at which C_max_ was observed

^d^AUC_0-last_ = Area under the curve, from 0 h to the last quantifiable timepoint

^e^MRT = mean residence time, or average time the molecule spends in circulation

**Fig 6:**
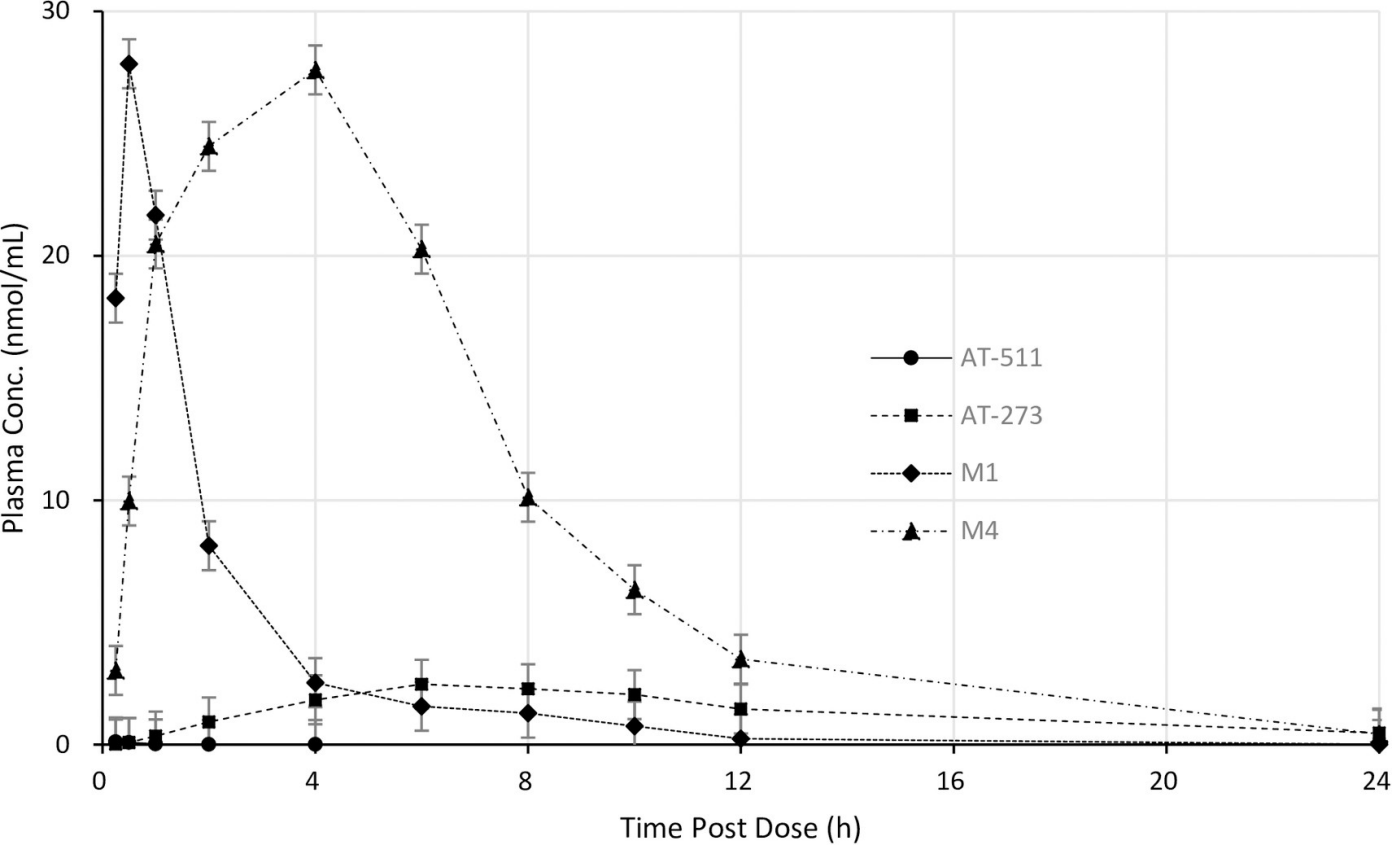
Mean profiles of AT-511 and its metabolites in rat plasma over 24 h. Sprague-Dawley rats (3 male and 3 female) were administered a 300 mg/kg single oral dose of AT-527 and plasma concentrations of AT-511, AT-273, M1 and M4 were measured by LC-MS/MS. Data are mean ± SD of all 6 animals.

In rats (n = 3 per sex), AT-511 was rapidly absorbed and efficiently converted to the L-alanyl metabolite, M1, as evidenced by their relative T_max_, C_max_ and AUC_0-last_ values (**Table 8, Fig 6, S4 Table**). The nucleoside metabolite of the MP still containing the N^6^-methyl modification, M4, peaked roughly 3 hours later. The nucleoside metabolite AT-273, which is formed from the dephosphorylation of AT-9010 and its DP and MP forms and is thus a measure of the intracellular formation of these phosphates, had the longest mean residence time (MRT; a measure of the average time a molecule spends in circulation) at 17.5 ± 2.1 h. Apart from M4 levels being somewhat higher in female rats, there were no obvious gender differences, so mean values (± SD) of the combined data from both sexes are presented.

**Fig 7:**
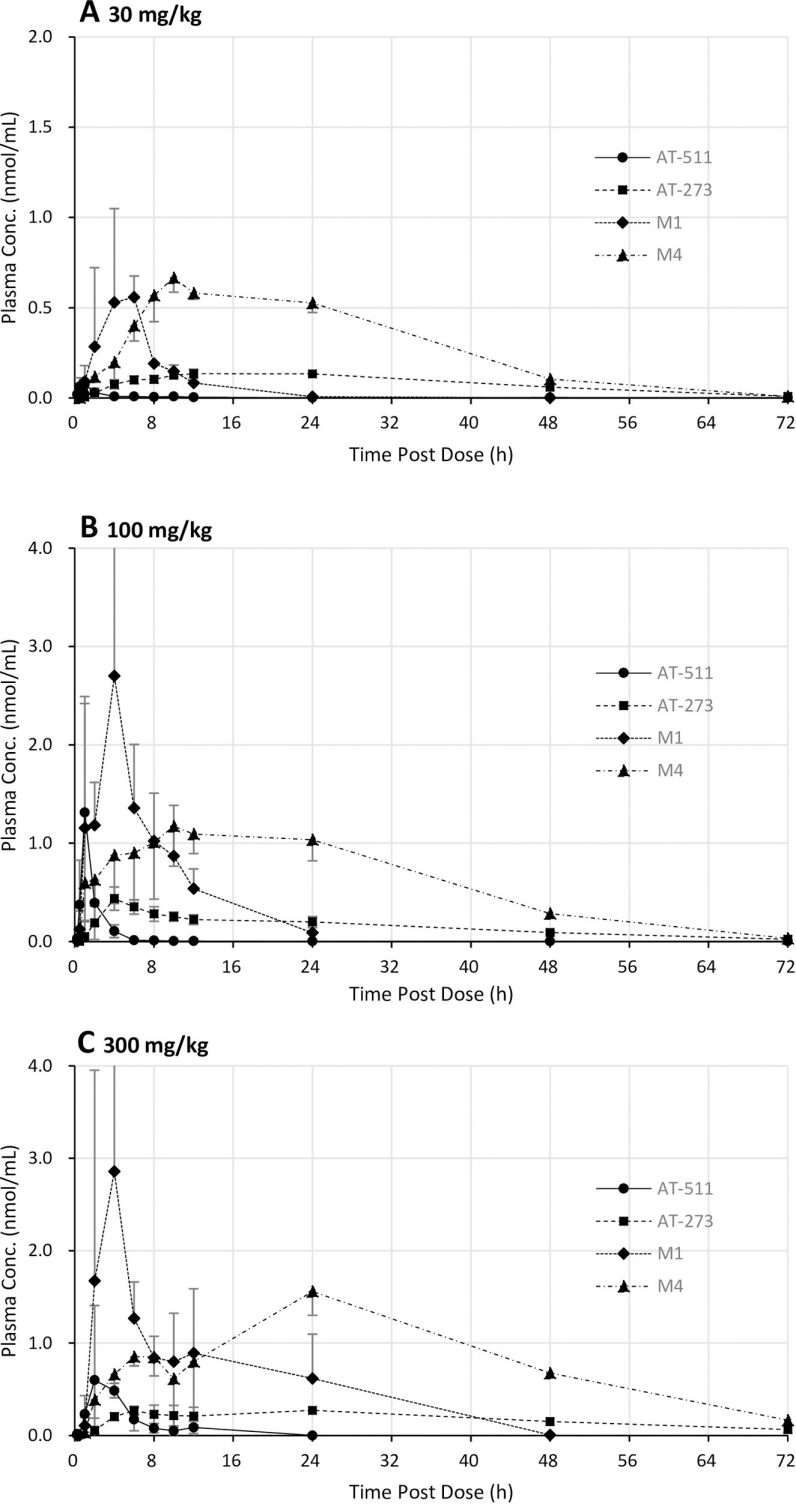
Mean profiles of AT-511 and its metabolites in monkey plasma over 72 h. Cynomolgus monkeys (groups of 3 males per dose) were administered a single oral dose of AT-527 at (A) 30 mg/kg, (B) 100 mg/kg and (C) 300 mg/kg, and plasma concentrations of AT-511, AT-273, M1 and M4 were measured by LC-MS/MS. Data are mean ± SD for each group.

Plasma profiles in male monkeys (n = 3 per dose) were similar to those in rats (**Fig 7**). Mean AUC_0-last_ values for AT-511 and M1 increased nearly dose proportionally but systemic exposures to M4 and AT-273 only rose 2- to 3-fold with the 10-fold increase in dose. This data reflects the rapid absorption and efficient conversion of AT-511 to M1, suggesting that elimination of the nucleoside metabolites M4 and AT-273 (likely via renal excretion) was not saturated as the dose was increased. Again, AT-273 had the longest MRT after administration of AT-527 at all three dose levels (**Table 8**), indicative of the long half-life of the active TP formed intracellularly.

### Preferential liver delivery

In rats (n = 2 per sex) 4 h after the 500 mg/kg oral dose of AT-527, the mean concentration of the active TP, AT-9010 (6974 ± 2844 pmol/g), was 87-fold higher than that in heart (80 ± 41 pmol/g). Similarly, mean liver/heart concentration ratios for AT-9010 in male monkeys 4 hr after an oral dose of AT-527 at 30, 100 or 300 mg/kg (n = 2 per dose) were 11–31 (**Table 9**), indicating preferential formation of the active TP in the liver of both species. In fact, in all heart tissues except one from a rat dosed at 500 mg/kg, concentrations of AT-9010 had to be estimated by extrapolation of the calibration curves below the lower limit of quantitation (LLOQ) of the LC-MS/MS assay (110 pmol/g). Moreover, as was observed in hepatocytes and Huh-7 cells, the active TP was the predominant metabolite measured in 4-h monkey livers at 30 and 100 mg/kg and its concentrations were dose proportional (545 ± 349 and 1890 ± 519 pmol/g, respectively) (**Table 9**). At the lower more therapeutically relevant dose of 30 mg/kg, the mean liver AT-9010 concentration was 34-fold higher than its parent phosphoramidate, again demonstrating efficient conversion of AT-511 to the active TP in liver cells. Monkey livers had 500-fold more AT-511 after the 300 mg/kg dose compared to the 30 mg/kg dose, suggesting that conversion to M1 via esterase activity becomes saturated at a very high dose. Nevertheless, even at this high dose, AT-9010 concentrations were 18 times higher in liver compared to corresponding heart tissues. In addition to the high liver/heart ratios for AT-9010 in monkeys, the similarly high ratios for its monophosphate precursors, M2 and M3 (11–72), are further evidence that these compounds are preferentially delivered to the liver, the target organ for HCV infection. As was observed in hepatocytes from all species tested, the 5’-TP of M2 was either not detected in monkey liver and heart tissue or possibly present but only at trace levels, well below the LLOQ of the assay (110 pmol/g).

**Table 9:** Mean (n = 2) liver and heart concentrations of AT-511 and its metabolites in male cynomolgus monkeys 4 h after administration of a single oral dose of AT-527.

Dose (mg/kg)	Analyte	Mean Concentration at 4 hr Postdose (pmol/g)		
		Liver	Heart	Liver/Heart Ratio
30	AT-511	15.0	7.8^a^	1.9^a^
	M1	319	86.3	3.7
	M2	103	5.4^a^	19^a^
	M3	78.8	7.5^a^	11^a^
	M4	381	180	2.1
	AT-273	267	88.5	3.0
	AT-9010	545	51.2^b^	11^b^
100	AT-511	70.3	14.3	4.9
	M1	671	187	3.6
	M2	479	9.9^a^	48^a^
	M3	203	7.9^a^	26^a^
	M4	1340	717	1.9
	AT-273	672	229	2.9
	AT-9010	1890	61.8^b^	31^b^
300	AT-511	7700	657	12
	M1	3510	630	5.6
	M2	87.9	6.6^a^	13^a^
	M3	1180	16.5	72
	M4	1950	1040	1.9
	AT-273	939	444	2.1
	AT-9010	968	55.3^b^	18^b^

^a^Means of heart concentrations contain values estimated by extrapolation of the calibration curve below the LLOQ (10–20 pmol/g) for all analytes except AT-9010

^b^Individual heart concentrations of AT-9010 were estimated by extrapolation of the calibration curve below the LLOQ of 110 pmol/g

The dose proportionality of AT-9010 in monkey livers between the 30 and 100 mg/kg doses was reflected in plasma levels of AT-273, with fold increases in respective C_max_ and AUC_0-last_ values of 3.1 and 1.9 with the 3.3-fold increase in dose. This is consistent with AT-273 only being formed via dephosphorylation of its phosphorylated forms (**Fig 2**). Since AT-9010 is the predominant intracellular phosphorylated metabolite, AT-273 can be considered a plasma marker of intracellular levels of the active triphosphate.

## Discussion

AT-511, the free base of the new drug candidate, AT-527, was significantly more potent *in vitro* against all HCV genotypes tested when compared to SOF, the only currently approved nucleoside NS5B inhibitor for treatment of HCV infections. Compared to SOF, AT-511 is 11–14 times more potent against the difficult-to-treat GT3. Moreover, AT-511 is 40–58 times more potent than SOF against RAVs containing the S282T polymorphism and at least 10-fold more potent than PSI-938, suggesting that AT-511 may potentially demonstrate greater clinical antiviral activity than these other two nucleotide prodrugs. This result is consistent with the high intracellular formation of the active triphosphate metabolite, AT-9010, in both Huh-7 cells and in human hepatocytes. The *in vivo* monkey and rat studies demonstrated that AT-511 was efficiently absorbed when dosed orally as its hemi-sulfate salt, AT-527, and was extensively converted to its active TP in liver, the target of HCV infection. Despite the relatively low concentrations of AT-511 in rat plasma after oral administration of AT-527, an observation which is consistent with the known high levels of esterase activity in rodent blood [16, 17], this species produces one of the highest amounts of the active TP in hepatocyte incubations *in vitro* as well as liver concentrations *in vivo*. Additionally, the rat and monkey studies showed that oral administration of a single dose of AT-527 resulted in preferential delivery of AT-9010 and its monophosphate precursors, M2 and M3 to the liver, and that the active TP was the predominant intracellular phosphorylated metabolite formed.

The potent antiviral activity of AT-511 appears to be relatively specific to HCV, since the drug showed only weak or no measurable activity against a panel of other viruses. The lack of activity against the DNA viruses (HSV-1, adenovirus and HBV) and HIV-1 (which contains an RNA genome that requires reverse transcription into DNA) may be attributed to the dissimilarity in the polymerases involved in the replication of these viruses compared to that of HCV (a positive-sense, single-stranded RNA virus). The apparent specificity of AT-511 against the other single-stranded RNA viruses tested, however, suggests that there are differences in the polymerases of these related viruses that render them relatively resistant to inhibition by AT-9010. Nevertheless, the potency of AT-511 across all HCV genotypes tested speaks to the highly conserved nature of the HCV NS5B genomic region.

Since certain N^6^- and O^6^-alkylated purines are substrates for ADALP1 only when an unsubstituted 5’-MP group is present [10], it is unlikely that the N^6^-methyl moiety of either M1 or M4 can be converted directly to their corresponding guanosine analog. Similarly, AT-273 can likely only be formed via dephosphorylation of its mono-, di- and tri-phosphorylated forms. The active TP is formed and retained intracellularly and its levels cannot be directly measured in blood samples. However, as AT-9010 is the most abundant metabolite formed in Huh-7 cells and hepatocytes *in vitro* and in liver tissue *in vivo*, plasma levels of AT-273 can be regarded as a surrogate of the intracellular levels of the active TP. Thus, the 10 h half-life of AT-9010 observed in human hepatocytes *in vitro* as well as the prolonged MRT of AT-273 in plasma of rats and monkeys after administration of a single oral dose of AT-527 are consistent with once daily oral dosing for treatment of HCV infections.

Arnold and colleagues [11] have reported that all antiviral ribonucleoside TP analogs tested, including SOF TP, were incorporated by POLRMT to some extent. Whereas mitochondrial toxicity has not been reported for SOF, the rapid development of cardiac toxicity observed in clinical trials with BMS-986094 [18] has been attributed to the ability of its active TP to inhibit POLRMT at concentrations 25- to 50-fold lower than those required of the TPs of AT-511 and SOF, as was demonstrated herein and previously reported for BMS-986094 and SOF [11]. Undoubtedly, the 2’-α-fluoro substitution in AT-511 and SOF that replaces the more naturally occurring 2’-α-hydroxy group in BMS-986094 is the reason for the far lower substrate efficiencies of the TPs of AT-511 and SOF for POLRMT compared to that of the TP of BMS-986094.

To assess the potential for AT-511 to cause mitochondrial toxicity, multiple *in vitro* tests were conducted. The glucose-galactose cytotoxicity assay in K562 cells did not detect any changes in mitochondrial function. Although this assay has been widely used to detect mitochondrial toxicity, several investigators have found it to be of limited value as some known mitochondrial toxins such as ddC did not generate cytotoxicity in galactose-adapted cells [19]. However, the lack of toxicity observed with AT-511 in the mitobiogenesis assay conducted in PC3 cells is reassuring as these cells are sensitive to mitochondrial toxicity and the mitochondrial protein synthesis assay is well-accepted for evaluating nucleoside analogs [12]. Moreover, AT-511 was not cytotoxic to iPS cardiomyocytes or bone marrow progenitor cells, indicating that this drug will not have the cardiotoxicity or myelosuppressive effects observed with other nucleoside analogs [19].

Other prodrugs of the monophosphate M3 were previously under development, including the phosphoramidate prodrug of O6-methylguanosine MP, PSI-661 [13], and the cyclic MP prodrug of O6-ethylguanosine, PSI-938 [14]. Despite the potent clinical anti-HCV activity of the latter drug, development of these compounds was discontinued, with PSI-938 halted due to hepatic toxicity observed with extended dosing in the clinic [8, 19]. These early prodrugs share a similar modification on the nucleobase and are subject to the same metabolic pathway leading to formation of mutagenic metabolites with methyl or ethyl substitutions at the O^6^ position of guanine [15, 20, 21]. AT-527 possesses a unique structural feature with an N6-methyl group that critically differentiates AT-527 from PSI-661 and PSI-938, with respect to metabolism. The results of the rhCYP3A4 study highlight this difference since, unlike PSI-661 and PSI-938, no mutagenic O^6^-alkylguanine bases were formed from AT-511, and only 1.8% was converted to other modified nucleobases, thus mitigating the hepatotoxicity risk from AT-527. Moreover, with almost no inhibitory effect on a panel of cytochrome P450 enzymes, AT-527 appears to have a low potential for metabolic drug-drug interactions.

With potent pan-genotypic antiviral activity and a favorable preclinical safety profile compared to other highly inhibitory protides of GTP analogs, AT-527 has the potential to be a more effective component of HCV therapies by achieving high SVR rates in all patient populations regardless of genotype and stage of disease with possibly shortened treatment duration. This would greatly improve ease of use and burden on patients with chronic HCV infection.

## Materials and methods

### Materials

Batches of AT-511 and AT-527 were provided by Atea Pharmaceuticals, Inc. and were solubilized in DMSO at 40 mM. Atea also provided AT-9010, and the active 5’-triphosphate metabolites of SOF (2’-F-2’-C-methyl UTP) and BMS-986094 (2’-C-methyl GTP) as 10 mM aqueous solutions for the mitochondrial assays, 5 mM BMS-986094, PSI-938, PSI-661, and reference standards for the cytochrome P450 metabolism studies, and the various 5’-mono- and triphosphate reference standards for assessment of their levels in liver and heart tissues. Test compounds in the various *in vitro* antiviral and selectivity assays were evaluated using top test concentrations of either 100, 20 or 1 μM and five serial dilutions in triplicate. The approved anti-HCV nucleoside analog, SOF (purchased from Sigma Aldrich, St. Louis, MO), was compared to AT-511 in several studies. Unless otherwise stated, positive control compounds were obtained from Sigma Aldrich.

### Methods

#### *In vitro* antiviral assays

*Huh-7 replicons harboring HCV GT1b NS3-NS5B coding sequences*. Huh-luc/neo-ET cells, harboring the persistently replicating I_389_luc-ubi-neo/NS3-3’/ET replicon containing the firefly luciferase gene-ubiquitin-neomycin phosphotransferase fusion protein and EMCV IRES driven NS3-5B HCV coding sequences containing the ET tissue culture adaptive mutations (E1202G, T1208I and K1846T) were seeded into 96-well plates (7.5x10^3^ cells/well), and incubated in DMEM supplemented with 10% FCS, 2 mM glutamine, penicillin (100 IU/mL)/streptomycin (100 μg/mL) and 1x nonessential amino acids plus 1 mg/mL G418 antibiotic at 37°C in 5% CO_2_ for 24 h. Media was then replaced with the same media minus G418 plus the test compounds in triplicate. Six wells in each plate received media alone as a no-treatment control. The cells were incubated an additional 72 h, and anti-HCV activity measured using the Britelite plus luminescence reporter gene kit according to the manufacturer’s instructions (Perkin Elmer, Shelton, CT). Duplicate plates were treated and incubated in parallel for assessment of cellular toxicity by XTT staining. The resulting data was used to determine the test article concentration required to inhibit virus replication by 50% (EC_50_) or 95% (EC_95_) and to reduce cell viability by 50% (TC_50_).

*PhenoSense HCV NS5B assay*. This assay, developed by Monogram Biosciences (San Francisco, CA), was used to determine the susceptibility of various HCV replicon resistance test vectors (RTVs) to AT-511 and the positive control, SOF. RTVs containing the NS5B coding regions of HCV prepared from laboratory strains, as well as strains derived from patients (clinical isolates), with identified genotypes and resistance-associated variants (RAVs), were used to quantitatively measure differences in inhibitor susceptibility relative to the drug susceptible reference replicon, MB_GT1b_Con1_NS5B (Con1). Preparation of RTV RNA is described in **S1 Protocol**. RTV RNA was electroporated into Huh-7 cells and incubated in the presence and absence of inhibitors at 4 and 72–96 h. Replication capacity was determined by evaluating luciferase activity at the later time point in the absence of inhibitor and relative to the control reference replicon RTV, HCV genotype 1b (Con1). A replication defective Con1 replicon was utilized to determine assay background. The percent inhibition at each serially diluted inhibitor concentration was derived as follows:

[1–(luciferaseactivityinthepresenceofinhibitor÷luciferaseactivityintheabsenceofinhibitor)]x100

Inhibitor susceptibility curves were derived from these values and inhibition data was interpolated and reported as fold-change relative to that of the reference. Cell viability was determined using the alamarBlue^®^ assay according to the manufacturer’s instructions (Invitrogen, Carlsbad, CA).

*Other antiviral assays*. The different cells and viruses were prepared as specified in **S2 Protocol**. Each assay was run in microtiter plates containing cell control wells (cells only), virus control wells (cells + virus), drug toxicity wells (cells + drug only), drug background wells (drug only) and experimental wells (drug or positive control + cells + virus). Samples were tested in triplicate with five half-log dilutions per compound. After cells, viruses and drugs were added, the plates were incubated at 37°C in 5% CO_2_, and the antiviral and cell viability assays conducted as described in **S2 Protocol**.

#### *In vitro* metabolism assays

*Huh-7 replicon cells*. The Huh-7 replicons harboring the HCV GT1b NS3-NS5B coding sequences used for the HCV inhibition assay were incubated in 6-well plates (1.7x10^5^ cells/well) with AT-511 at 10 μM for 0, 2, 4, 6, 8 and 24 h. At each time point, the media was removed and the cells were washed twice with cold PBS, collected in 1 mL cold 60% MeOH, and extracted overnight at -20°C with internal standard added. After centrifugation, at 10,000 *g* and 4°C for 10 min, the supernatant was analyzed for concentrations of AT-511 and its metabolites by LC-MS/MS as described in **S3 Protocol**. Untreated cells were collected at each time point as a negative control.

*Hepatocytes*. Plated cryopreserved hepatocytes from humans (mixed gender, pool of 10 donors), male Sprague Dawley rat and male cynomolgus monkey were purchased from Sekisui XenoTech (Kansas City, KS), plated with Matrigel overlay in 6- or 24-well plates, and maintained overnight at 37°C in 5% CO_2_ with medium containing pen-strep antibiotics (XenoTech). Cryopreserved male mouse (CD1), and fresh male Beagle dog and male cynomolgus monkey hepatocytes were obtained from BioreclamationIVT (Baltimore, MD) and were similarly plated with Matrigel in 6- or 24-well plates and maintained overnight in *InVitro*GRO^™^HI medium with Torpedo^™^ antibiotic (BioreclamationIVT). The protocol described above for measuring AT-511 and its metabolites in Huh-7 cells was repeated with the five different hepatocytes. Untreated cells were collected at 24 h, and the protein content of the cell lysates were determined using a bicinchoninic acid protein assay kit to assess the number of cells in each incubation. Separately, plated human hepatocytes were incubated for 6 h with 100 μM AT-511, followed by washout periods of 0, 2, 6, 18 and 30 h. Cells were washed, lysed in 1 mL cold 60% MeOH overnight at -20°C, and the lysates collected to determine the half-life of the active metabolite, AT-9010 by LC-MS/MS as described for the analysis of triphosphates in **S3 Protocol**.

#### HCV NS5B polymerase (RdRp) assay

RdRp activity was determined by the incorporation of radiolabeled ribonucleotide into synthesized RNA. The 50μl RdRp reaction mixture, which contained 25mM Tris-HCl, pH 7.5, 5mM MgCl_2_, 25mM KCl, 17mM NaCl, 3mM DTT, 1μM UTP, 48nM *in vitro* transcribed RNA template that is complementary to HCV (-) strand 3'UTR region, 1μCi ^3^H-UTP, 250 μM rCTP, 1 or 250 μM rATP, rGTP (the reduced amount should give a positive inhibitory response for the particular nucleotide analog being evaluated) and enzyme (derived from GT1b) prepared by Southern Research (Frederick, MD), was added to serially diluted AT-9010 in 96-well plates. After 2 h incubation and extensive washing, radioactivity, measured by a microplate scintillation counter, was used to determine the potency of compound inhibition. HCV796 (Southern Research, Frederick, MD) was used as positive control for the assay.

#### Human DNA polymerase inhibition assay

The polymerases were purchased from CHIMERx (Madison, WI). Inhibition of DNA polymerase α activity was assayed in microtiter plates in 20 mM Tris-HCl, pH 8, 5 mM Mg acetate, 0.3 mg/mL BSA, 1mM DTT, 0.1 mM spermine, 0.05 mM dCTP, dTTP and dATP, 10 μCi ^32^P-α-dGTP (800 Ci/mmol), 20 μg activated calf thymus DNA and the test compound at indicated concentrations. The DNA polymerase β and γ reaction mixture contained 50 mM Tris-HCl, pH 8.7, KCl (10 mM for β and 100 mM for γ), 10 mM MgCl_2_, 0.4 mg/mL BSA, 1mM DTT, 15% glycerol, 0.05 mM dCTP, dTTP and dATP, 10 μCi ^32^P-α-dGTP (800 Ci/mmol), 20 μg activated calf thymus DNA and test compound at the indicated concentrations. For each assay, samples were run in triplicate, and positive controls were included. The enzyme reactions were allowed to proceed for 30 min at 37ºC followed by transfer onto glass-fiber filter plates and subsequent precipitation with 10% trichloroacetic acid (TCA). Plates were washed five times with 5% TCA, once with 95% ethanol and dried. The radioactive content of each filter was measured and used to determine the extent of inhibition of each polymerase.

#### POLRMT nucleotide incorporation assay

The nucleoside triphosphates (NTP) tested were AT-9010 (for AT-527), 2’-F-2’-C-methyl UTP (for SOF) and 2’-C-methyl GTP (for BMS-986094). GTP and UTP were used as positive controls. Single nucleotide incorporation and chain termination experiments were conducted and analyzed as described by Cameron et al [11].

#### Cytotoxicity *a*ssays

*Cardiomyocyte assay*. Human iPS cardiomyocytes (Cellular Dynamics; Madison, WI), in medium from Cellular Dynamics, were seeded in 96-well plates (1.5x10^4^ cells/well) and incubated at 37°C in 5% CO_2_ for 48 h. Test or positive control compounds were then added in triplicate and the incubations continued for 3 days. Cell viability was measured by XTT and CellTiter Glo staining. For the latter method, the medium was removed from test plates, replaced with CellTiter Glo reagent in fresh medium before incubating at RT for 10 min. The well contents were transferred to a white 96-well plate and luminescence measured within 15 min on a Wallac 1450 Microbeta Trilux liquid scintillation counter.

*Bone marrow progenitor cell inhibition assays*. Bone marrow progenitor cells, suspended in 6-well plates (1x10^5^ cells/well) in Iscove modified Dulbecco medium containing 15% heat-inactivated FBS, 10% giant cell tumor conditioned medium (Bone Marrow Plus, Sigma), 10 ng/mL recombinant human IL-6, 10 ng/mL recombinant human IL-3, 25 ng/mL recombinant human granulocyte macrophage colony stimulating factor (GM-CSF, R&D systems), and 1% methylcellulose, were incubated with test or positive control compounds at 37°C in 5% CO_2_ for 14 days. The resulting number of colonies (greater than 30 cells) were compared to control wells to determine the extent of cytotoxicity.

*Mitochondrial toxicity assay*. K562 leukemia cells (ATCC, CCL-243) seeded in 96-well plates (1x10^5^ cells/well) were incubated in triplicate with test or positive control compounds in RPMI containing glucose, 10% FBS and 2 mM L-glutamine. Plates were incubated at 37°C in 5% CO_2_ for 3 days, and cell viability measured by XTT staining. Viability was compared with untreated cells to determine TC_50_ values. Separately, K562 cells were incubated in triplicate with test compounds in glucose-free RPMI supplemented with 10 mM galactose. Test compounds diluted in RPMI containing glucose were evaluated in parallel. Following incubation at 37°C in 5% CO_2_ for 2 h, differential biomarkers associated with changes in membrane integrity for necrosis were measured by fluorescence (485/520 nm), and cellular adenosine triphosphate (ATP) levels for mitochondrial dysfunction were measured by chemiluminescence using the specific reagents provided in the Promega Mitochondrial ToxGlo kit (Madison, WI).

*Mitobiogenesis toxicity assay*. PC3 cells (prostate adenocarcinoma; ATCC, CRL-1435) seeded in 96-well plates (2.5x10^3^ cells/well) were incubated at 37°C in 5% CO_2_ for 5 days in triplicate with test or positive control compounds in DMEM supplemented with 10% FBS, 2 mM L-glutamine, 100 U/mL penicillin and 100 μg/mL streptomycin. One plate was used to measure cell viability by XTT staining. Cells in the remaining two plates were fixed with 4% paraformaldehyde for 20 min, washed thrice with DPBS, and 0.5% acetic acid added for 5 min to block endogenous alkaline phosphatase activity. Nuclear DNA encoded Complex II SDH-A mitochondrial protein and mitochondrial encoded Complex IV COX-1 protein were measured spectrophotometrically using the Abcam Mitobiogenesis ELISA kit (Cambridge, UK) per the manufacturer’s instructions.

#### Cytochrome P450 studies

*Metabolism by rhCYP3A4*. AT-511 (5 μM) was incubated in triplicate at 37°C with 50 nM rhCYP3A4 with cytochrome b5 in the presence of an NADPH regenerating system containing 1.3 mM NADP^+^, 3.3 mM glucose-6-phosphate, 3.3 mM MgCl2 and 0.4 U/mL glucose-6-phosphate dehydrogenase (G6PDH) in 100 mM potassium phosphate buffer, pH 7.4, for 0 and 2 h. Samples were then extracted by the addition of 2 volumes of ice-cold quench solution (10% ACN in MeOH) containing proper internal standards, and analyzed by LC/MS and LC/MS/MS for the formation of potential metabolites. BMS-986094, PSI-938 and PSI-661 were similarly evaluated. Time 0 samples and reactions without rhCYP3A4 served as negative controls, while reactions replacing the test compounds with midazolam (2 μM, BD Biosciences, Franklin Lakes, NJ), were positive controls.

*Inhibition of P450 isozymes*. AT-511 (0.01–30 μM) was incubated in duplicate with human liver microsomes in the presence of 2 mM NADPH in 100 mM potassium phosphate (pH 7.4) containing 5 mM MgCl2 and a probe substrate (S3 Table). Selective CYP inhibitors were screened as positive controls (S3 Table). Except for CYP2C9 (15 min) and CYP2C19 (60 min), samples were incubated for 10 min at 37°C, and the reactions terminated by addition of MeOH containing the internal standard propranolol. The quenched samples were kept on ice for 10 min and centrifuged at 4°C for 10 min. The supernatant was removed and the probe substrate metabolites were analyzed by LC-MS/MS. A decrease in the formation of the metabolite compared to vehicle control was used to calculate IC_50_ values [22].

#### Animal studies

*Animal welfare*. All animal studies described herein were conducted at WuXi AppTec (Shanghai, China) in strict compliance with AAALAC International and NIH guidelines as described in the “Guide for the Care and Use of Laboratory Animals”, National Research Council–ILAR, Revised 2011 and the People’s Republic of China, Ministry of Science and Technology, “Regulations for the Administration of Affairs Concerning Experimental Animals”, 2017. All studies were conducted in full compliance with protocols that were reviewed and approved by WuXi AppTec’s Institutional Animal Care and Use Committee (IACUC) prior to study initiation (IACUC numbers R20151119-Rat and SZ20150908-Monkeys-4), and all animals were assessed as to their general health by a member of the WuXi AppTec veterinary staff upon arrival and prior to being placed on study. All animals were housed in rooms with controlled temperature (18 to 26°C), relative humidity (30 to 70%) and light cycle (12 h artificial light and 12 h dark), with 10 to 20 air changes/hr and were provided with manipulatives/enrichment toys.

*Rat pharmacokinetic (PK) study*. Sprague-Dawley rats were obtained from SLAC Laboratory Animal Co., Ltd. (Shanghai, China), group housed (during the 3-day acclimation period) or individually housed (during the study) and provided ad libitum access to purified (reverse osmosis; RO) water and food except when fasted overnight prior to administration of test article, 4 h after which access to food was restored. Two groups of five rats (single sex/group) were administered a single dose of 500 mg/kg AT-527, suspended in 0.5% CMC-Na and 0.5% Tween 80, via nasogastric gavage followed by a vehicle flush of 3 mL (about 3 times the nasogastric tube volume). This dosage was selected because it was close to the anticipated highest dose to be used in a repeated oral dose toxicity study and would permit accurate assessments of plasma concentrations of metabolites. Blood samples (200 μL) were collected via the saphenous vein using manual restraint at 0.25, 0.5, 1, 2, 4, 6, 8, 10, 12, 24, 48 and 72 h post dose from three animals per sex, and at 0.25, 0.5, 1, 2 and 4 h post dose from two animals per sex. Blood samples were transferred to tubes containing 4 μL 0.5 M dipotassium ethylenediaminetetraacetic acid (K_2_EDTA) and 10 μL dichlorvos (2 mg/mL) to prevent coagulation and inhibit esterase activity, respectively, mixed and centrifuged (3000 x g, 10 min, 2–8°C) to prepare plasma samples which were quickly frozen on dry ice and stored at ≤ -80°C until LC-MS/MS analysis. Animals were observed for any unusual or adverse clinical signs just before and immediately after dosing and prior to each blood collection time point, with no such signs noted. After the 4-h blood collection, two rats per group were euthanized by CO_2_ inhalation followed by rapid excision and snap-freezing of the heart and liver in liquid nitrogen. The frozen organs were broken into small pieces and approximately 0.5 g of each tissue was homogenized in 5 volumes (w/v) pre-chilled homogenization solution (30% 268 mM EDTA adjusted to pH 7.2–7.4 with potassium hydroxide in MeOH) in a dry ice-ethanol bath. Samples of each homogenate were extracted with 4 volumes of internal standard solution 1 (ISS1; 200 ng/mL dexamethasone, 200 ng/mL tolbutamide, 200 ng/mL ((2R,3S,5R,Z)-5-(2,4-dioxo-3,4-dihydropyrimidin-1(2H)-yl)-4-(1-fluoroethylidene)-3-hydroxytetrahydrofuran-2-yl)methyl tetrahydrogen triphosphate and 40 mM dibutyl acetic acid (DBAA) in MeOH) and supernatants were clarified by centrifugation (13000 rpm, 10 min, 4°C). Concentrations of AT-511, AT-273, M1, and M4 in plasma samples, and AT-9010 in heart and liver homogenate supernatants were determined by LC-MS/MS.

*Monkey PK study*. Male non-naïve cynomolgus monkeys were obtained from Hainan Jingang Laboratory Animal Co., Ltd. (Hainan, China), group housed during the 5-day acclimation period or individually housed during the study in stainless steel mesh cages, provided ad libitum access to RO water, fed twice daily (~120 g Certified Monkey Diet daily; Beijing Vital Keao Feed Co., Ltd., Beijing, China) and given daily treats of fresh fruit. Additional environmental enrichment included classical music and cartoons. Three groups of 5 monkeys each were administered 30, 100 or 300 mg/kg AT-527 as a powder filled in empty porcine hard gelatin capsules (size 0). The monkeys were fasted overnight prior to dosing and fed 4 h after dosing. Three dosages were selected to provide assessments of dose proportionality, the top two representing potential doses for a repeated oral dose toxicity study in monkeys, and the lowest predicted to be more therapeutically relevant. A pill gun was used to place the capsule beyond the root of the animals’ tongues and their mouths were held loosely closed for approximately 30 sec to allow them to move the jaw and swallow the capsule. Immediately following capsule administration, 20 mL of water was given by mouth and then the mouth was opened to ensure the capsule was swallowed. Blood samples (~0.6 mL) were collected up to 72 h from three animals per dose group, and up to 4 h from the remaining two animals in each group, at the same time points described for rats. Animals were observed for any unusual or adverse clinical signs just before and immediately after dosing and prior to each blood collection time point, with no such signs noted. Blood samples were collected with a syringe from a peripheral blood vessel from restrained, non-sedated animals, immediately transferred to pre-chilled tubes containing ~1 mg K_2_EDTA•2H_2_0 and 20 μL of 5 mM dichlorvos solution, mixed and placed on ice until plasma was prepared by centrifugation as described for rats. Immediately after centrifugation, plasma samples were extracted with 4 volumes ISS2, and the supernatants were clarified by centrifugation (12,000 rpm, 10 min, 4°C) and stored at ≤ -60°C until analyzed by LC-MS/MS as described below. After the 4-hr blood collection, two monkeys per dose group were anesthetized with intravenous injection of pentobarbital sodium (60 mg/kg) and then euthanized as described for rats. Samples (~1 g) of liver and heart tissue were collected from each animal at the same organ location, snap frozen in liquid nitrogen and homogenate supernatants were prepared as described for rat tissues except one replicate of each tissue homogenate was extracted with 4 volumes of internal standard solution 2 (ISS2; 100 ng/mL labetalol, 100 ng/mL dexamethasone, 100 ng/mL tolbutamide, 100 ng/mL verapamil, 100 ng/mL glyburide and 100 ng/mL celecoxib in MeOH:ACN, 3:1, v/v) for determining concentrations of AT-511, AT-273, M1, M2, M3 and M4 and another replicate was extracted with 4 volumes of ISS1 for quantitating AT-9010 determining concentrations of AT-9010. All analytes were quantitated by LC-MS/MS as described below.

*LC-MS/MS analysis of rat and monkey plasma and tissue homogenate supernatants*. For the analysis of AT-511 in rat plasma, aliquots were extracted with 10 volumes ISS2. The supernatants were clarified by centrifugation (4000 rpm for 10 min at 4°C) and 3 μL samples were injected onto an Acuity BEH C18 1.7 μm, 2.1 × 50 mm column and a Sciex API 4000 mass spectrometer (ESI positive ion, MRM mode). Mobile phases A (0.025% formic acid (FA) and 1 mM ammonium acetate (AA) in 5% ACN in water) and B (0.025% FA and 1 mM AA in 95% ACN in water) were used to elute samples (3 μL) at 0.6 mL/min with 10% B for 0.3 min followed by linear gradients to 60% B over 0.6 min and to 90% B over 0.2 min, and ending with 90% B for 0.4 min. The analysis of AT-273, M1 and M4 in rat plasma was the same except samples were extracted with 4 volumes of ISS2, 6 μL samples were injected onto a Gemini C18 5 μm, 4.6 x 50 mm column and a Sciex QTRAP 6500, mobile phase A was 0.3% FA in water, and samples (6 μL) were eluted at 0.8 mL/min with 2% B for 0.8 min followed by linear gradients to 30% B over 1.4 min and 98% B over 1.4 min and ending with 98% B for 1.4 min. The analysis of AT-511, AT-273, M1 and M4 in monkey plasma was conducted the same as the analysis of AT-273, M1 and M4 in rat plasma except freshly prepared plasma samples were extracted prior to storage as described and 5 μL aliquots were analyzed by LC-MS/MS.

For the analysis of AT-9010 in rat tissue homogenate supernatant, aliquots were diluted 1:2 with water and injected onto an Agilent Zorbax Extend C18 5 μm, 2.1 × 50 mm column and a Sciex API 4000 mass spectrometer (ESI positive ion, MRM mode). Mobile phases A (0.001% ammonia and 0.18 mM DBAA in water (1:1, v/v) and B (10 mM dimethylhexylamine (DMHA) and 3 mM AA in 50% ACN in water) were used to elute samples (60 μL) at 0.7 mL/min with 2% B for 0.8 min followed by a linear gradient to 30% B over 0.4 min, isocratic elution at 30% B for 0.4 min, a linear gradient to 95% B over 0.4 min, and ending with 95% B for 0.4 min. The analysis of AT-9010 in monkey tissue homogenate supernatant was the same except the sample injection volume was 20 μL. Attempts to measure tissue levels of other potentially formed TPs, including 2,6-diaminopurine-2’-F-2’-C-methylribosyl-5’-triphosphate and 2-amino-6-methylaminopurine-2’-F-2’-C-methylribosyl-5’-triphosphate (M2-TP), also used this method. The analysis of AT-511, AT-273, M1 and M4 in monkey tissue homogenate supernatant was the same as for the analysis of these analytes in monkey plasma except supernatants were diluted 1:3 with water prior to analysis using a Sciex API 4000 or 6500 mass spectrometer.

For the analysis of M2 and M3 in monkey tissue homogenate supernatant, aliquots were diluted 1:3 with water and injected onto an Acuity HSS T3 1.8 μm, 2.1 × 50 mm column and a Sciex API 6500 mass spectrometer (ESI positive ion, MRM mode). Mobile phases A (0.3% FA and 10 mM AA in water and B (0.3% FA and 10 mM AA in 95% ACN in water) were used to elute samples (1 μL) at 0.6 mL/min with 0% B for 0.4 min followed by linear gradients to 30% B over 0.5 min and to 95% B over 0.2 min, ending with isocratic elution at 95% B for 0.4 min.

*PK data analysis*. Plasma concentrations of AT-511, AT-273, M1 and M4 from animals that were not used for tissue collection (3 rats/sex/dose group or 3 male monkeys/dose group) were subjected to non-compartmental pharmacokinetic analysis using Phoenix WinNonlin software (version 6.3, Pharsight, Mountain View, CA). The linear/log trapezoidal rule was applied in obtaining the PK parameters.

## Supporting information

S1 Table Antiviral activity and cellular toxicity of AT-511 and SOF assayed in parallel in HCV 1b replicons. (DOCX)

S2 Table Inhibition of cytochrome P450 isoforms by AT-511 and positive controls. (DOCX)

S3 Table Inhibition of cytochrome P450 isoforms by AT-511 and positive controls. (DOCX)

S4 Table Individual and mean plasma concentrations (nmol/mL) of AT-511 and AT-273 in Sprague-Dawley rats used for plasma pharmacokinetic parameter determinations following single oral administration of AT-527 at 500 mg/kg. (DOCX)

S5 Table Individual and mean plasma concentrations (nmol/mL) of M1 and M4 in Sprague-Dawley rats used for plasma pharmacokinetic parameter determinations following single oral administration of AT-527 at 500 mg/kg. (DOCX)

S6 Table Individual and mean plasma concentrations (nmol/mL) of AT-511 and AT-273 in male cynomolgus monkeys following single oral administration of AT-527 at 30 mg/kg. (DOCX)

S7 Table Individual and mean plasma concentrations (nmol/mL) of M1 and M4 in male cynomolgus monkeys following single oral administration of AT-527 at 30 mg/kg. (DOCX)

S8 Table Individual and mean plasma concentrations (nmol/mL) of AT-511 and AT-273 in male cynomolgus monkeys following single oral administration of AT-527 at 100 mg/kg. (DOCX)

S9 Table Individual and mean plasma concentrations (nmol/mL) of M1 and M4 in male cynomolgus monkeys following single oral administration of AT-527 at 100 mg/kg. (DOCX)

S10 Table Individual and mean plasma concentrations (nmol/mL) of AT-511 and AT-273 in male cynomolgus monkeys following single oral administration of AT-527 at 300 mg/kg. (DOCX)

S11 Table Individual and mean plasma concentrations (nmol/mL) of M1 and M4 in male cynomolgus monkeys following single oral administration of AT-527 at 300 mg/kg. (DOCX)

S1 Fig SOF TP (AT9001) incorporation catalyzed by POLRMT. (DOCX)

S2 Fig AT-9010 (AT9010) incorporation catalyzed by POLRMT. (DOCX)

S3 Fig BMS-986094 TP (AT9002) incorporation catalyzed by POLRMT. (DOCX)

S1 Protocol Preparation of HCV RTV RNA. (DOCX)

S2 Protocol Non-HCV antiviral assays. (DOCX)

S3 Protocol LC-MS/MS analysis of AT-511 and metabolites in cultured cells. (DOCX)
