# Supplementary material for: Preclinical evaluation of AT-527, a novel guanosine nucleotide prodrug with potent, pan-genotypic activity against hepatitis C virus
Source: PLoS One. 2020 Jan 8;15(1):e0227104. doi: 10.1371/journal.pone.0227104 (PMC6949113; doi:10.1371/journal.pone.0227104)
Supplement: S1 Table — (DOCX) [file pone.0227104.s001.docx]

**S1 Table. Antiviral activity and cellular toxicity of AT-511 and sofosbuvir (SOF) assayed in parallel in HCV 1b replicons**

| **Assay #** | **AT-511 Batch #** | **AT-511** | | | **SOF** | | |
| --- | --- | --- | --- | --- | --- | --- | --- |
|  |  | **EC_50_ (nM)** | **EC_95_ (nM)** | **TC_50_ (nM)** | **EC_50_ (nM)** | **EC_95_ (nM)** | **TC_50_ (nM)** |
| 1 | 1 | 11.5 | 50.0 | >1000 | 67.5 | 387 | >1000 |
| 2 | 1 | 4.68 | 28.3 | >1000 | 32.7 | 197 | >1000 |
| 3 | 1 | 4.09 | 26.7 | >1000 | 48.3 | 247 | >1000 |
| 4 | 2 | 3.53 | 20.3 | >100 | 40.4 | 198 | >1000 |
| 5 | 3 | 6.14 | 26.2 | >1000 | 47.6 | 275 | >1000 |
| 6 | 3 | 4.93 | 28.9 | >100 | 48.1 | 263 | >1000 |
| 6 | 4 | 3.73 | 30.3 | >1000 | as above | as above | >1000 |
| 7 | 5 | 0.78 | 2.99 | >100000 | 52.3 | 360 | >1000 |
| 8 | 6 | 2.41 | 9.61 | >100 | 46.5 | 231 | >1000 |
| Mean | N/A | 4.6 | 24.8 | N/A | 47.9 | 270 | N/A |
| SD | N/A | 3.0 | 13.4 | N/A | 10.0 | 70.1 | N/A |
| range | N/A | 0.78-11.5 | 2.99-50.0 | N/A | 32.7-67.5 | 197-387 | N/A |

Antiviral activity was measured using the Britelite assay and cytotoxicity was determined by XTT

staining as described in the Methods.

EC_50_, effective concentration required to inhibit virus replication by 50%

EC_95_, effective concentration required to inhibit virus replication by 95%

TC_50_, threshold concentration required to decrease cell viability by 50%
