# Supplementary material for: Preclinical evaluation of AT-527, a novel guanosine nucleotide prodrug with potent, pan-genotypic activity against hepatitis C virus
Source: PLoS One. 2020 Jan 8;15(1):e0227104. doi: 10.1371/journal.pone.0227104 (PMC6949113; doi:10.1371/journal.pone.0227104)
Supplement: S2 Table — (DOCX) [file pone.0227104.s002.docx]

**S2 Table. Concentrations of potential nucleobase metabolites after 2-h incubations of PSI-661, PSI-938, BMS-986094 or AT-511 with recombinant human cytochrome P450 3A4**

| **Test**  **Article** | **Guanine** | | **O^6^-Methyl-**  **guanine** | | **O^6^-Ethyl-**  **guanine** | | **2,6-Diamino-**  **purine** | | **N^6^-Methyl-2,6-diaminopurine** | |
| --- | --- | --- | --- | --- | --- | --- | --- | --- | --- | --- |
|  | **Conc. (µM)** | **% of Starting Conc.** | **Conc. (µM)** | **% of Starting Conc.** | **Conc. (µM)** | **% of Starting Conc.** | **Conc. (µM)** | **% of Starting Conc.** | **Conc. (µM)** | **% of Starting Conc.** |
| PSI-661 | ND | <0.5^a^ | 2.56  ±0.057 | 51.2 | ND | <0.5^a^ | ND | <0.5^a^ | ND | <0.5^a^ |
| PSI-938 | ND | <0.5^a^ | ND | <0.5^a^ | 1.57  ±0.06 | 31.4 | ND | <0.5^a^ | ND | <0.5^a^ |
| BMS-986094 | ND | <0.5^a^ | 0.434  ±0.007 | 8.6 | ND | <0.5^a^ | ND | <0.5^a^ | ND | <0.5^a^ |
| AT-511 | ND | <0.3^b^ | ND^a^ | <0.3^b^ | ND | <0.3^b^ | 0.065  ±0.010 | 1.3 | 0.024  ±0.006 | 0.5 |

Test articles at a starting concentration of 5 µM were incubated in triplicate with rhCYP3A4 and NADPH for 2 h and concentrations of five potential nucleobase metabolites were determined by LC-MS/MS. The resulting amounts of each nucleobase are reported as mean concentration ± standard deviation and as percent of the starting concentration of each test article.

ND, not detected; well below the lower limit of quantitation

^a^Lower limit of quantitation was 0.025 µM

^b^Lower limit of quantitation was 0.015 µM
