# Supplementary material for: Preclinical evaluation of AT-527, a novel guanosine nucleotide prodrug with potent, pan-genotypic activity against hepatitis C virus
Source: PLoS One. 2020 Jan 8;15(1):e0227104. doi: 10.1371/journal.pone.0227104 (PMC6949113; doi:10.1371/journal.pone.0227104)
Supplement: S3 Table — (DOCX) [file pone.0227104.s003.docx]

**S3 Table.** **Inhibition of cytochrome P450 isoforms by AT-511 and positive controls**

| **P450 Isoform** | **Probe Substrate** | **Substrate Conc. (µM)** | **HLM Conc. (mg/mL)** | **Incubation Time (min)** | **Positive Control Inhibitor** | **IC_50_ (µM)** | |
| --- | --- | --- | --- | --- | --- | --- | --- |
|  |  |  |  |  |  | **Positive Control** | **AT-511** |
| CYP1A2 | Tacrine | 5 | 0.2 | 10 | α-Naphthoflavone | 0.012 | >30 |
| CYP2B6 | Bupropion | 100 | 0.25 | 10 | Ticlopidine | 0.317 | >30 |
| CYP2C8 | Amodiaquine | 5 | 0.25 | 10 | Quercetin | 4.61 | >30 |
| CYP2C9 | Tolbutamide | 100 | 0.5 | 15 | Sulfaphenazole | 0.128 | >30 |
| CYP2C19 | Mephenytoin | 100 | 0.25 | 60 | Ticlopidine | 1.22 | >30 |
| CYP2D6 | Dextromethorphan | 5 | 0.5 | 10 | Quinidine | 0.051 | >30 |
| CYP3A4 | Midazolam | 2.5 | 0.25 | 10 | Ketoconazole | 0.014 | 26.1 |
| CYP3A4 | Testosterone | 50 | 0.25 | 10 | Ketoconazole | 0.020 | 24.6 |

Inhibition was measured as described in the Methods.

HLM, human liver microsomes

IC_50_, concentration of inhibitor required to achieve 50% inhibition
