# Supplementary material for: Preclinical evaluation of AT-527, a novel guanosine nucleotide prodrug with potent, pan-genotypic activity against hepatitis C virus
Source: PLoS One. 2020 Jan 8;15(1):e0227104. doi: 10.1371/journal.pone.0227104 (PMC6949113; doi:10.1371/journal.pone.0227104)
Supplement: S4 Table — (DOCX) [file pone.0227104.s004.docx]

**S4 Table. Individual and mean plasma concentrations (nmol/mL) of AT-511 and AT-273 in Sprague-Dawley rats used for plasma pharmacokinetic parameter determinations following single oral administration of AT-527 at 500 mg/kg**

| **Analyte** | **Time (h)** | **Male Rat Number** | | | **Female Rat Number** | | | **Mean** | **SD** |
| --- | --- | --- | --- | --- | --- | --- | --- | --- | --- |
|  |  | **1** | **2** | **3** | **7** | **8** | **9** |  |  |
| AT-511 | 0.250 | 0.104 | 0.124 | 0.086 | 0.101 | 0.201 | 0.039 | 0.109 | 0.053 |
|  | 0.500 | 0.048 | 0.042 | 0.077 | 0.067 | 0.154 | 0.111 | 0.083 | 0.042 |
|  | 1.00 | 0.030 | 0.101 | 0.010 | 0.023 | 0.006 | 0.006 | 0.029 | 0.037 |
|  | 2.00 | 0.006 | BQL | 0.023 | 0.025 | BQL | BQL | ND | ND |
|  | 4.00 | BQL | 0.015 | 0.004 | BQL | BQL | 0.004 | ND | ND |
|  | 6.00 | BQL | BQL | BQL | BQL | BQL | BQL | ND | ND |
|  | 8.00 | 0.007 | BQL | BQL | BQL | BQL | BQL | ND | ND |
|  | 10.0 | 0.002 | BQL | BQL | BQL | BQL | BQL | ND | ND |
|  | 12.0 | BQL | BQL | BQL | BQL | BQL | BQL | ND | ND |
|  | 24.0 | BQL | BQL | BQL | BQL | BQL | BQL | ND | ND |
|  | 48.0 | BQL | BQL | BQL | BQL | BQL | BQL | ND | ND |
|  | 72.0 | BQL | BQL | BQL | BQL | BQL | BQL | ND | ND |
|  | 0.250 | 0.025 | 0.025 | 0.027 | 0.023 | 0.025 | 0.019 | 0.024 | 0.003 |
|  | 0.500 | 0.100 | 0.087 | 0.095 | 0.094 | 0.083 | 0.072 | 0.088 | 0.010 |
|  | 1.00 | 0.272 | 0.330 | 0.303 | 0.368 | 0.371 | 0.468 | 0.352 | 0.068 |
|  | 2.00 | 0.992 | 0.882 | 0.772 | 0.882 | 0.912 | 1.133 | 0.929 | 0.122 |
|  | 4.00 | 1.788 | 1.487 | 1.380 | 1.971 | 1.757 | 2.619 | 1.834 | 0.441 |
| AT-273 | 6.00 | 2.105 | 1.610 | 1.537 | 3.127 | 2.472 | 3.976 | 2.471 | 0.943 |
|  | 8.00 | 1.874 | 1.781 | 1.323 | 2.676 | 2.299 | 3.775 | 2.288 | 0.863 |
|  | 10.0 | 1.981 | 1.266 | 1.193 | 2.315 | 2.105 | 3.408 | 2.045 | 0.809 |
|  | 12.0 | 1.463 | 1.129 | 0.936 | 1.437 | 1.320 | 2.449 | 1.456 | 0.526 |
|  | 24.0 | 0.705 | 0.244 | 0.275 | 0.332 | 0.765 | 0.528 | 0.475 | 0.225 |
|  | 48.0 | 0.213 | 0.039 | 0.074 | 0.093 | 0.171 | 0.274 | 0.144 | 0.090 |
|  | 72.0 | 0.127 | 0.193 | 0.043 | 0.118 | 0.146 | 0.070 | 0.116 | 0.053 |

BQL, below the quantifiable limit of 0.0017 nmol/mL for AT-511 and 0.0032 nmol/mL for AT-273
ND, not determined as more than half of the individual values were not quantifiable
