# Supplementary material for: Preclinical evaluation of AT-527, a novel guanosine nucleotide prodrug with potent, pan-genotypic activity against hepatitis C virus
Source: PLoS One. 2020 Jan 8;15(1):e0227104. doi: 10.1371/journal.pone.0227104 (PMC6949113; doi:10.1371/journal.pone.0227104)
Supplement: S5 Table — (DOCX) [file pone.0227104.s005.docx]

**S5 Table. Individual and mean plasma concentrations (nmol/mL) of M1 and M4 in Sprague-Dawley rats used for plasma pharmacokinetic parameter determinations following single oral administration of AT-527 at 500 mg/kg**

| **Analyte** | **Time (h)** | **Male Rat Number** | | | **Female Rat Number** | | | **Mean** | **SD** |
| --- | --- | --- | --- | --- | --- | --- | --- | --- | --- |
|  |  | **1** | **2** | **3** | **7** | **8** | **9** |  |  |
| M1 | 0.250 | 11.178 | 19.896 | 17.307 | 22.443 | 19.810 | 18.947 | 18.264 | 5.961 |
|  | 0.500 | 14.286 | 25.896 | 20.760 | 41.001 | 25.896 | 39.275 | 27.852 | 16.170 |
|  | 1.00 | 15.818 | 21.558 | 14.955 | 24.601 | 13.746 | 39.275 | 21.659 | 14.861 |
|  | 2.00 | 6.129 | 3.712 | 4.683 | 13.811 | 3.776 | 16.703 | 8.136 | 8.762 |
|  | 4.00 | 2.395 | 2.119 | 2.849 | 2.611 | 2.132 | 3.107 | 2.536 | 0.614 |
|  | 6.00 | 1.929 | 2.080 | 1.709 | 1.610 | 0.643 | 1.446 | 1.570 | 0.785 |
|  | 8.00 | 3.582 | 1.388 | 0.240 | 1.109 | 0.391 | 1.006 | 1.286 | 1.869 |
|  | 10.0 | 1.647 | 0.440 | 0.213 | 1.034 | 0.360 | 0.833 | 0.754 | 0.827 |
|  | 12.0 | 0.129 | 0.091 | 0.036 | 0.399 | 0.322 | 0.432 | 0.235 | 0.264 |
|  | 24.0 | 0.005 | 0.003 | 0.002 | 0.005 | 0.013 | 0.018 | 0.008 | 0.010 |
|  | 48.0 | BQL | BQL | BQL | BQL | BQL | BQL | ND | ND |
|  | 72.0 | BQL | BQL | BQL | BQL | BQL | BQL | ND | ND |
| M4 | 0.250 | 2.411 | 2.622 | 2.651 | 3.426 | 4.259 | 2.824 | 3.032 | 0.693 |
|  | 0.500 | 6.372 | 8.678 | 6.852 | 13.256 | 12.872 | 11.752 | 9.964 | 3.057 |
|  | 1.00 | 11.752 | 17.707 | 11.976 | 23.311 | 24.848 | 33.301 | 20.482 | 8.339 |
|  | 2.00 | 13.673 | 13.865 | 10.887 | 34.582 | 25.488 | 48.351 | 24.474 | 14.745 |
|  | 4.00 | 18.252 | 10.311 | 12.648 | 37.144 | 30.259 | 56.996 | 27.602 | 17.732 |
|  | 6.00 | 8.934 | 8.998 | 9.254 | 31.220 | 20.621 | 42.587 | 20.269 | 14.107 |
|  | 8.00 | 6.564 | 5.796 | 3.874 | 14.505 | 10.439 | 19.533 | 10.118 | 5.971 |
|  | 10.0 | 5.443 | 2.568 | 2.626 | 9.734 | 6.212 | 11.431 | 6.336 | 3.641 |
|  | 12.0 | 2.315 | 1.569 | 1.540 | 4.259 | 4.995 | 6.276 | 3.492 | 1.974 |
|  | 24.0 | 1.175 | 0.050 | 0.230 | 0.148 | 0.512 | 0.445 | 0.427 | 0.407 |
|  | 48.0 | 0.067 | 0.009 | 0.009 | BQL | 0.038 | 0.102 | 0.037 | 0.040 |
|  | 72.0 | 0.016 | 0.026 | BQL | 0.024 | 0.020 | 0.022 | 0.018 | 0.009 |

BQL, below the quantifiable limit of 0.0022 nmol/mL for M1 and 0.0032 nmol/mL for M4
ND, not determined as more than half of the individual values were not quantifiable
