# Supplementary material for: Preclinical evaluation of AT-527, a novel guanosine nucleotide prodrug with potent, pan-genotypic activity against hepatitis C virus
Source: PLoS One. 2020 Jan 8;15(1):e0227104. doi: 10.1371/journal.pone.0227104 (PMC6949113; doi:10.1371/journal.pone.0227104)
Supplement: S6 Table — (DOCX) [file pone.0227104.s006.docx]

**S6 Table. Individual and mean plasma concentrations (nmol/mL) of AT-511 and AT-273 in male cynomolgus monkeys following single oral administration of AT-527 at 30 mg/kg**

| **Analyte** | **Time (h)** | **Monkey Number** | | | **Mean** | **SD** |
| --- | --- | --- | --- | --- | --- | --- |
|  |  | **1** | **2** | **3** |  |  |
| AT-511 | 0.250 | 0.022 | 0.020 | 0.020 | 0.021 | 0.001 |
|  | 0.500 | 0.020 | 0.111 | 0.060 | 0.064 | 0.046 |
|  | 1.00 | 0.023 | 0.042 | 0.164 | 0.076 | 0.076 |
|  | 2.00 | 0.053 | 0.009 | 0.029 | 0.030 | 0.022 |
|  | 4.00 | 0.008 | 0.013 | 0.003 | 0.008 | 0.005 |
|  | 6.00 | 0.006 | 0.018 | 0.002 | 0.009 | 0.008 |
|  | 8.00 | 0.005 | 0.009 | 0.003 | 0.005 | 0.003 |
|  | 10.0 | 0.014 | 0.007 | 0.002 | 0.008 | 0.006 |
|  | 12.0 | 0.005 | 0.006 | 0.002 | 0.004 | 0.002 |
|  | 24.0 | 0.002 | 0.002 | 0.002 | 0.002 | 0.000 |
|  | 48.0 | 0.002 | 0.002 | 0.003 | 0.002 | 0.000 |
|  | 72.0 | 0.002 | BQL | 0.002 | 0.001 | 0.001 |
| AT-273 | 0.250 | BQL | BQL | BQL | ND | ND |
|  | 0.500 | BQL | BQL | BQL | ND | ND |
|  | 1.00 | BQL | 0.008 | 0.008 | 0.006 | 0.005 |
|  | 2.00 | 0.014 | 0.025 | 0.041 | 0.027 | 0.013 |
|  | 4.00 | 0.094 | 0.068 | 0.067 | 0.076 | 0.015 |
|  | 6.00 | 0.103 | 0.125 | 0.071 | 0.100 | 0.027 |
|  | 8.00 | 0.096 | 0.131 | 0.082 | 0.103 | 0.025 |
|  | 10.0 | 0.115 | 0.151 | 0.115 | 0.127 | 0.021 |
|  | 12.0 | 0.116 | 0.157 | 0.131 | 0.135 | 0.020 |
|  | 24.0 | 0.138 | 0.141 | 0.122 | 0.134 | 0.010 |
|  | 48.0 | 0.062 | 0.084 | 0.034 | 0.060 | 0.025 |
|  | 72.0 | 0.008 | 0.012 | 0.004 | 0.008 | 0.004 |

BQL, below the quantifiable limit of 0.0017 nmol/mL for AT-511 and 0.0032 nmol/mL for AT-273
ND, not determined as more than half of the individual values were not quantifiable
