# Supplementary material for: Preclinical evaluation of AT-527, a novel guanosine nucleotide prodrug with potent, pan-genotypic activity against hepatitis C virus
Source: PLoS One. 2020 Jan 8;15(1):e0227104. doi: 10.1371/journal.pone.0227104 (PMC6949113; doi:10.1371/journal.pone.0227104)
Supplement: S7 Table — (DOCX) [file pone.0227104.s007.docx]

**S7 Table. Individual and mean plasma concentrations (nmol/mL) of M1 and M4 in male cynomolgus monkeys following single oral administration of AT-527 at 30 mg/kg**

| **Analyte** | **Time (h)** | **Monkey Number** | | | **Mean** | **SD** |
| --- | --- | --- | --- | --- | --- | --- |
|  |  | **1** | **2** | **3** |  |  |
| M1 | 0.250 | BQL | BQL | BQL | ND | ND |
|  | 0.500 | BQL | 0.006 | 0.008 | 0.005 | 0.004 |
|  | 1.00 | 0.003 | 0.060 | 0.211 | 0.091 | 0.107 |
|  | 2.00 | 0.252 | 0.216 | 0.384 | 0.284 | 0.089 |
|  | 4.00 | 0.319 | 1.034 | 0.237 | 0.530 | 0.438 |
|  | 6.00 | 0.425 | 1.131 | 0.118 | 0.558 | 0.519 |
|  | 8.00 | 0.145 | 0.324 | 0.103 | 0.191 | 0.117 |
|  | 10.0 | 0.143 | 0.145 | 0.154 | 0.148 | 0.006 |
|  | 12.0 | 0.047 | 0.085 | 0.116 | 0.083 | 0.035 |
|  | 24.0 | 0.005 | 0.009 | 0.008 | 0.007 | 0.002 |
|  | 48.0 | BQL | BQL | 0.002 | ND | ND |
|  | 72.0 | BQL | BQL | BQL | ND | ND |
| M4 | 0.250 | BQL | BQL | BQL | ND | ND |
|  | 0.500 | 0.000 | 0.000 | 0.003 | 0.001 | 0.002 |
|  | 1.00 | 0.000 | 0.025 | 0.122 | 0.049 | 0.065 |
|  | 2.00 | 0.102 | 0.037 | 0.215 | 0.118 | 0.090 |
|  | 4.00 | 0.167 | 0.231 | 0.196 | 0.198 | 0.032 |
|  | 6.00 | 0.359 | 0.528 | 0.316 | 0.401 | 0.112 |
|  | 8.00 | 0.474 | 0.589 | 0.640 | 0.568 | 0.085 |
|  | 10.0 | 0.532 | 0.647 | 0.820 | 0.666 | 0.145 |
|  | 12.0 | 0.522 | 0.551 | 0.672 | 0.582 | 0.080 |
|  | 24.0 | 0.538 | 0.554 | 0.487 | 0.526 | 0.035 |
|  | 48.0 | 0.104 | 0.157 | 0.051 | 0.104 | 0.053 |
|  | 72.0 | 0.009 | 0.013 | 0.004 | 0.009 | 0.005 |

BQL, below the quantifiable limit of 0.0022 nmol/mL for M1 and 0.0032 nmol/mL for M4
ND, not determined as more than half of the individual values were not quantifiable
