# Supplementary material for: Preclinical evaluation of AT-527, a novel guanosine nucleotide prodrug with potent, pan-genotypic activity against hepatitis C virus
Source: PLoS One. 2020 Jan 8;15(1):e0227104. doi: 10.1371/journal.pone.0227104 (PMC6949113; doi:10.1371/journal.pone.0227104)
Supplement: S8 Table — (DOCX) [file pone.0227104.s008.docx]

**S8 Table. Individual and mean plasma concentrations (nmol/mL) of AT-511 and AT-273 in male cynomolgus monkeys following single oral administration of AT-527 at 100 mg/kg**

| **Analyte** | **Time (h)** | **Monkey Number** | | | **Mean** | **SD** |
| --- | --- | --- | --- | --- | --- | --- |
|  |  | **1** | **2** | **3** |  |  |
| AT-511 | 0.250 | 0.023 | 0.026 | 0.049 | 0.033 | 0.014 |
|  | 0.500 | 0.025 | 0.208 | 0.887 | 0.374 | 0.454 |
|  | 1.00 | 0.162 | 2.373 | 1.400 | 1.312 | 1.108 |
|  | 2.00 | 0.265 | 0.657 | 0.251 | 0.391 | 0.230 |
|  | 4.00 | 0.102 | 0.172 | 0.042 | 0.105 | 0.065 |
|  | 6.00 | 0.005 | 0.010 | 0.023 | 0.013 | 0.010 |
|  | 8.00 | 0.005 | 0.017 | 0.007 | 0.010 | 0.007 |
|  | 10.0 | 0.005 | 0.006 | 0.006 | 0.006 | 0.000 |
|  | 12.0 | 0.004 | 0.004 | 0.004 | 0.004 | 0.000 |
|  | 24.0 | 0.002 | 0.002 | 0.002 | 0.002 | 0.000 |
|  | 48.0 | 0.002 | 0.002 | BQL | 0.002 | 0.001 |
|  | 72.0 | 0.002 | BQL | 0.002 | 0.001 | 0.001 |
| AT-273 | 0.250 | BQL | BQL | BQL | ND | ND |
|  | 0.500 | BQL | BQL | 0.005 | ND | ND |
|  | 1.00 | 0.007 | 0.025 | 0.120 | 0.051 | 0.061 |
|  | 2.00 | 0.086 | 0.122 | 0.364 | 0.190 | 0.152 |
|  | 4.00 | 0.333 | 0.344 | 0.631 | 0.436 | 0.169 |
|  | 6.00 | 0.298 | 0.271 | 0.488 | 0.352 | 0.118 |
|  | 8.00 | 0.250 | 0.228 | 0.364 | 0.280 | 0.073 |
|  | 10.0 | 0.230 | 0.194 | 0.337 | 0.254 | 0.075 |
|  | 12.0 | 0.210 | 0.185 | 0.274 | 0.223 | 0.046 |
|  | 24.0 | 0.255 | 0.182 | 0.159 | 0.199 | 0.050 |
|  | 48.0 | 0.142 | 0.032 | 0.099 | 0.091 | 0.055 |
|  | 72.0 | 0.027 | 0.005 | 0.040 | 0.024 | 0.017 |

BQL, below the quantifiable limit of 0.0022 nmol/mL for M1 and 0.0032 nmol/mL for M4
ND, not determined as more than half of the individual values were not quantifiable
