# Supplementary material for: Preclinical evaluation of AT-527, a novel guanosine nucleotide prodrug with potent, pan-genotypic activity against hepatitis C virus
Source: PLoS One. 2020 Jan 8;15(1):e0227104. doi: 10.1371/journal.pone.0227104 (PMC6949113; doi:10.1371/journal.pone.0227104)
Supplement: S9 Table — (DOCX) [file pone.0227104.s009.docx]

**S9 Table. Individual and mean plasma concentrations (nmol/mL) of M1 and M4 in male cynomolgus monkeys following single oral administration of AT-527 at 100 mg/kg**

| **Analyte** | **Time (h)** | **Monkey Number** | | | **Mean** | **SD** |
| --- | --- | --- | --- | --- | --- | --- |
|  |  | **1** | **2** | **3** |  |  |
| M1 | 0.250 | BQL | BQL | 0.004 | ND | ND |
|  | 0.500 | 0.004 | 0.024 | 0.341 | 0.123 | 0.189 |
|  | 1.00 | 0.166 | 0.619 | 2.676 | 1.154 | 1.337 |
|  | 2.00 | 0.805 | 1.085 | 1.659 | 1.183 | 0.436 |
|  | 4.00 | 4.985 | 1.101 | 2.016 | 2.700 | 2.031 |
|  | 6.00 | 1.757 | 0.606 | 1.703 | 1.355 | 0.649 |
|  | 8.00 | 0.783 | 0.701 | 1.582 | 1.022 | 0.486 |
|  | 10.0 | 0.725 | 0.438 | 1.442 | 0.868 | 0.517 |
|  | 12.0 | 0.598 | 0.313 | 0.701 | 0.537 | 0.201 |
|  | 24.0 | 0.200 | 0.039 | 0.025 | 0.088 | 0.097 |
|  | 48.0 | BQL | BQL | 0.002 | ND | ND |
|  | 72.0 | BQL | BQL | BQL | ND | ND |
| M4 | 0.250 | BQL | BQL | BQL | ND | ND |
|  | 0.500 | BQL | 0.007 | 0.146 | 0.051 | 0.082 |
|  | 1.00 | 0.085 | 0.320 | 1.377 | 0.594 | 0.688 |
|  | 2.00 | 0.391 | 0.429 | 1.063 | 0.628 | 0.378 |
|  | 4.00 | 0.842 | 0.688 | 1.102 | 0.877 | 0.209 |
|  | 6.00 | 0.775 | 0.484 | 1.441 | 0.900 | 0.491 |
|  | 8.00 | 0.823 | 0.653 | 1.553 | 1.010 | 0.478 |
|  | 10.0 | 0.893 | 0.788 | 1.838 | 1.173 | 0.578 |
|  | 12.0 | 0.852 | 0.868 | 1.563 | 1.094 | 0.406 |
|  | 24.0 | 1.198 | 0.813 | 1.095 | 1.035 | 0.199 |
|  | 48.0 | 0.419 | 0.037 | 0.394 | 0.283 | 0.214 |
|  | 72.0 | 0.033 | BQL | 0.065 | 0.033 | 0.033 |

BQL, below the quantifiable limit of 0.0022 nmol/mL for M1 and 0.0032 nmol/mL for M4
ND, not determined as more than half of the individual values were not quantifiable
