# Supplementary material for: Preclinical evaluation of AT-527, a novel guanosine nucleotide prodrug with potent, pan-genotypic activity against hepatitis C virus
Source: PLoS One. 2020 Jan 8;15(1):e0227104. doi: 10.1371/journal.pone.0227104 (PMC6949113; doi:10.1371/journal.pone.0227104)
Supplement: S10 Table — (DOCX) [file pone.0227104.s010.docx]

**S10 Table. Individual and mean plasma concentrations (nmol/mL) of AT-511 and AT-273 in male cynomolgus monkeys following single oral administration of AT-527 at 300 mg/kg**

| **Analyte** | **Time (h)** | **Monkey Number** | | | **Mean** | **SD** |
| --- | --- | --- | --- | --- | --- | --- |
|  |  | **1** | **2** | **3** |  |  |
| AT-511 | 0.250 | BQL | 0.004 | 0.047 | 0.017 | 0.026 |
|  | 0.500 | BQL | 0.007 | 0.006 | 0.004 | 0.004 |
|  | 1.00 | 0.022 | 0.265 | 0.414 | 0.234 | 0.198 |
|  | 2.00 | 0.094 | 0.174 | 1.531 | 0.600 | 0.807 |
|  | 4.00 | 0.561 | 0.495 | 0.404 | 0.487 | 0.079 |
|  | 6.00 | 0.315 | 0.130 | 0.080 | 0.175 | 0.124 |
|  | 8.00 | 0.123 | 0.088 | 0.026 | 0.079 | 0.049 |
|  | 10.0 | 0.101 | 0.039 | 0.013 | 0.051 | 0.045 |
|  | 12.0 | 0.153 | 0.088 | 0.022 | 0.087 | 0.065 |
|  | 24.0 | 0.004 | BQL | BQL | ND | ND |
|  | 48.0 | BQL | BQL | BQL | ND | ND |
|  | 72.0 | BQL | BQL | BQL | ND | ND |
| AT-273 | 0.250 | BQL | BQL | BQL | ND | ND |
|  | 0.500 | BQL | BQL | BQL | ND | ND |
|  | 1.00 | BQL | BQL | BQL | ND | ND |
|  | 2.00 | 0.024 | 0.051 | 0.092 | 0.056 | 0.034 |
|  | 4.00 | 0.134 | 0.188 | 0.291 | 0.204 | 0.080 |
|  | 6.00 | 0.262 | 0.260 | 0.285 | 0.269 | 0.014 |
|  | 8.00 | 0.230 | 0.263 | 0.197 | 0.230 | 0.033 |
|  | 10.0 | 0.245 | 0.231 | 0.169 | 0.215 | 0.041 |
|  | 12.0 | 0.258 | 0.211 | 0.163 | 0.211 | 0.047 |
|  | 24.0 | 0.368 | 0.280 | 0.170 | 0.273 | 0.099 |
|  | 48.0 | 0.266 | 0.144 | 0.045 | 0.152 | 0.111 |
|  | 72.0 | 0.174 | 0.024 | BQL | 0.066 | 0.094 |

BQL, below the quantifiable limit of 0.0017 nmol/mL for AT-511 and 0.0032 nmol/mL for AT-273
ND, not determined as more than half of the individual values were not quantifiable
