# Supplementary material for: Preclinical evaluation of AT-527, a novel guanosine nucleotide prodrug with potent, pan-genotypic activity against hepatitis C virus
Source: PLoS One. 2020 Jan 8;15(1):e0227104. doi: 10.1371/journal.pone.0227104 (PMC6949113; doi:10.1371/journal.pone.0227104)
Supplement: S11 Table — (DOCX) [file pone.0227104.s011.docx]

**S11 Table. Individual and mean plasma concentrations (nmol/mL) of M1 and M4 in male cynomolgus monkeys following single oral administration of AT-527 at 300 mg/kg**

| **Analyte** | **Time (h)** | **Monkey Number** | | | **Mean** | **SD** |
| --- | --- | --- | --- | --- | --- | --- |
|  |  | **1** | **2** | **3** |  |  |
| M1 | 0.250 | BQL | BQL | 0.004 | ND | ND |
|  | 0.500 | BQL | 0.002 | 0.003 | 0.002 | 0.002 |
|  | 1.00 | 0.008 | 0.132 | 0.182 | 0.107 | 0.090 |
|  | 2.00 | 0.154 | 0.576 | 4.294 | 1.675 | 2.278 |
|  | 4.00 | 0.432 | 1.321 | 6.819 | 2.857 | 3.460 |
|  | 6.00 | 1.027 | 1.055 | 1.722 | 1.268 | 0.393 |
|  | 8.00 | 0.973 | 0.572 | 0.978 | 0.841 | 0.233 |
|  | 10.0 | 0.783 | 0.285 | 1.331 | 0.800 | 0.524 |
|  | 12.0 | 0.883 | 0.202 | 1.593 | 0.892 | 0.695 |
|  | 24.0 | 0.455 | 0.237 | 1.157 | 0.616 | 0.480 |
|  | 48.0 | 0.015 | BQL | BQL | ND | ND |
|  | 72.0 | BQL | BQL | BQL | ND | ND |
| M4 | 0.250 | BQL | BQL | BQL | ND | ND |
|  | 0.500 | BQL | BQL | BQL | ND | ND |
|  | 1.00 | BQL | 0.054 | 0.036 | 0.030 | 0.028 |
|  | 2.00 | 0.084 | 0.207 | 0.871 | 0.387 | 0.423 |
|  | 4.00 | 0.394 | 0.493 | 1.098 | 0.662 | 0.381 |
|  | 6.00 | 0.644 | 0.877 | 1.041 | 0.854 | 0.200 |
|  | 8.00 | 0.797 | 1.025 | 0.724 | 0.849 | 0.157 |
|  | 10.0 | 0.532 | 0.727 | 0.586 | 0.615 | 0.101 |
|  | 12.0 | 0.768 | 1.018 | 0.615 | 0.801 | 0.204 |
|  | 24.0 | 1.969 | 1.908 | 0.797 | 1.558 | 0.660 |
|  | 48.0 | 1.607 | 0.371 | 0.051 | 0.677 | 0.822 |
|  | 72.0 | 0.461 | 0.032 | BQL | 0.164 | 0.257 |

BQL, below the quantifiable limit of 0.0022 nmol/mL for M1 and 0.0032 nmol/mL for M4
ND, not determined as more than half of the individual values were not quantifiable
