# Supplementary material for: Preclinical evaluation of AT-527, a novel guanosine nucleotide prodrug with potent, pan-genotypic activity against hepatitis C virus
Source: PLoS One. 2020 Jan 8;15(1):e0227104. doi: 10.1371/journal.pone.0227104 (PMC6949113; doi:10.1371/journal.pone.0227104)
Supplement: S1 Fig — (DOCX) [file pone.0227104.s012.docx]

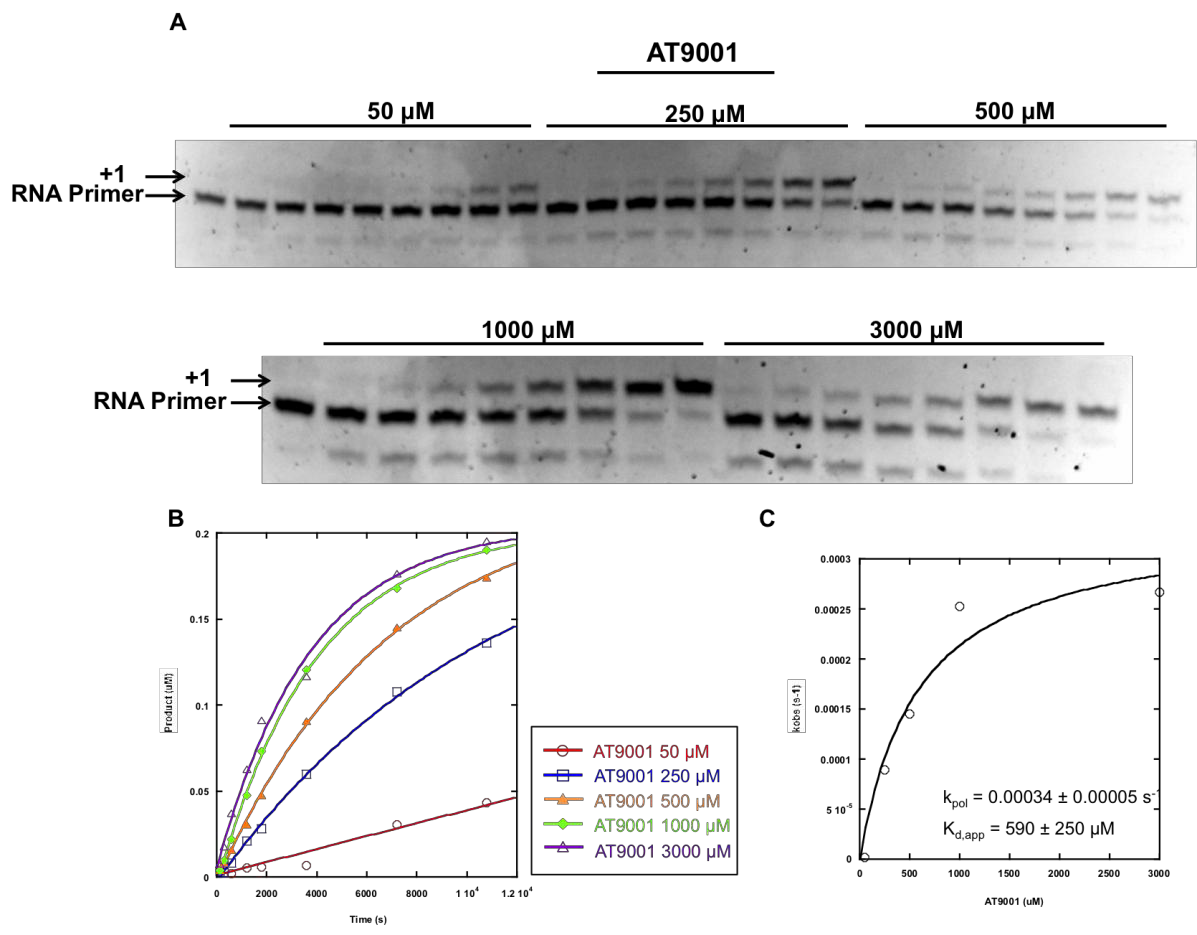
**S1 Fig. SOF TP (AT9001) incorporation catalyzed by POLRMT. (A)** POLRMT (0.5 µM) was incubated with fluorescein-labeled-RNA/DNA scaffold (0.2 µM) for 1 min and then rapidly mixed with SOF TP (50-3000 µM). Reactions were quenched at various times with EDTA (50 µM). **(B)** Quantitated RNA product was plotted as a function of time and fit to a single exponential equation. **(C)** Values for k_obs_ were plotted as a function of SOF TP concentration and fit to as hyperbola, yielding a k_pol_ value of 0.00034 ± 0.00005 s^-1^ and a K_d,app_ value of 590 ± 250 µM).
