# Supplementary material for: Preclinical evaluation of AT-527, a novel guanosine nucleotide prodrug with potent, pan-genotypic activity against hepatitis C virus
Source: PLoS One. 2020 Jan 8;15(1):e0227104. doi: 10.1371/journal.pone.0227104 (PMC6949113; doi:10.1371/journal.pone.0227104)
Supplement: S3 Fig — (DOCX) [file pone.0227104.s014.docx]

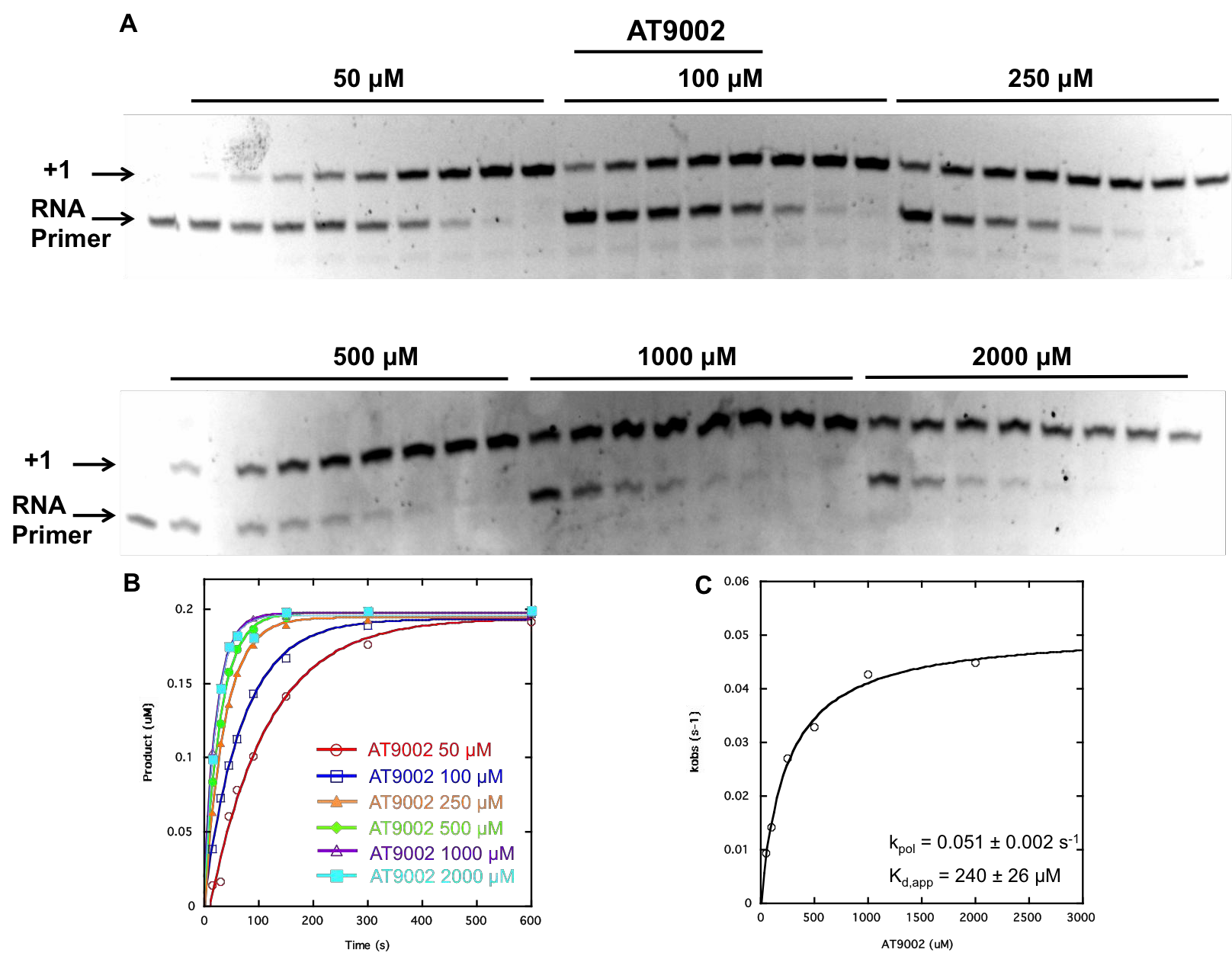


**S3 Fig. BMS-986094 TP (AT9002) incorporation catalyzed by POLRMT. (A)** POLRMT (0.5 µM) was incubated with fluorescein-labeled-RNA/DNA scaffold (0.2 µM) for 1 min and then rapidly mixed with BMS-986094 TP (50-2000 µM). Reactions were quenched at various times with EDTA (50 µM). **(B)** Quantitated RNA product was plotted as a function of time and fit to a single exponential equation. **(C)** Values for k_obs_ were plotted as a function of BMS-986094 TP concentration and fit to as hyperbola, yielding a k_pol_ value of 0.051 ± 0.002 s^-1^ and a K_d,app_ value of 240 ± 26 µM).
