# Supplementary material for: Preclinical evaluation of AT-527, a novel guanosine nucleotide prodrug with potent, pan-genotypic activity against hepatitis C virus
Source: PLoS One. 2020 Jan 8;15(1):e0227104. doi: 10.1371/journal.pone.0227104 (PMC6949113; doi:10.1371/journal.pone.0227104)
Supplement: S1 Protocol — (DOCX) [file pone.0227104.s015.docx]

**S1 Protocol. Preparation of HCV RTV RNA**

**Sample Preparation and Amplification.** Virus particles in 200 μL aliquots of frozen samples were disrupted by the addition of lysis buffer containing a chaotropic agent. Genomic viral RNA (vRNA) was extracted from viral lysates using oligonucleotide-linked magnetic beads. Purified vRNA was used as a template for first-strand cDNA synthesis in a reverse transcriptase (RT) reaction. The resulting cDNA was used as the template for the first round of a nested polymerase chain reaction (PCR) that resulted in the amplification of the entire NS5B region. Due to the sequence variation between subtypes 1a and 1b, specific 1a and 1b RT and first- and second-round PCR primers were used.

**Cloning Patient Derived NS5B Regions into the RTV.** The second-round (nested) PCR amplification primer set contained restriction endonuclease recognition/cleavage sites that enabled cloning of NS5B amplification products into an HCV replicon RTV for phenotypic drug susceptibility analysis. PCR products were purified by agarose gel electrophoresis and subsequent column chromatography to remove residual primers, primer-dimers and non-specific reaction products and were then subjected to restriction endonuclease digestion. The digestion reaction was purified using column chromatography and the amplification product was then ligated into a luciferase reporter replicon RTV. Ligation reactions were used to transform competent *E. coli*. Plasmid DNA was purified from bacterial cultures, using silica column chromatography, and was quantified by spectrophotometry.

**Preparation of RTV RNA**. Prior to *in vitro* transcription of the RTV, the plasmid DNA template was linearized by restriction endonuclease digestion and was column purified. The RTV contained hepatitis delta virus ribozyme sequences for appropriate termination of replicon RNA following *in vitro* transcription. *In vitro* transcribed RNA was column purified, quantified and the integrity was evaluated using electrophoretic separation.
