# Supplementary material for: Preclinical evaluation of AT-527, a novel guanosine nucleotide prodrug with potent, pan-genotypic activity against hepatitis C virus
Source: PLoS One. 2020 Jan 8;15(1):e0227104. doi: 10.1371/journal.pone.0227104 (PMC6949113; doi:10.1371/journal.pone.0227104)
Supplement: S2 Protocol — (DOCX) [file pone.0227104.s016.docx]

**S2 Protocol. Non-HCV Antiviral Assays**

**Anti-Human Immunodeficiency Virus (HIV-1).** CEM-SS cells were obtained from the NIH AIDS Research and Reference Reagent Program (Manassas, VA), resuspended at 5x10^4^ cells per mL and 50 μL added to the microtiter plate wells. The lymphocyte-tropic virus strain HIV-1_RF_ (obtained from the NIH AIDS Research and Reference Reagent Program and grown in CEM-SS cells for stock virus pools) was used for the assay. Virus was diluted into tissue culture medium (RPMI1640 supplemented with 10% heat-inactivated fetal bovine serum, 2 mM L-glutamine, 100 U/mL penicillin and 100 µg/mL streptomycin) such that the amount of virus added to each well in a volume of 50 μL was the amount determined to yield 85 to 95% cell killing at 6 days post-infection. Antiviral activity and cytotoxicity were determined by XTT staining as described below.

**Anti-Zika Virus (Zika).** Vero cells (African green monkey kidney, ATCC CCL-81) grown in DMEM supplemented with 10% FBS, 2 mM L-glutamine, 100 U/mL penicillin and 10 μg/mL streptomycin were resuspended at 5x10^4^ cells per mL and 100 μL added to each well. The microtiter plates were then incubated at 37°C in 5% CO_2_ overnight to allow for cell adherence. Monolayers were approximately 70% confluent for the assay. Zika virus strain MR766 was obtained from ATCC (VR-84) and a stock virus pool was produced in LLCMK2 cells. Virus was diluted into the assay medium (DMEM supplemented with 2% heat-inactivated FBS, 2 mM L-glutamine, 100 U/mL penicillin and 10 μg/mL streptomycin) such that the amount of virus added to each well in a volume of 100 μL was the amount determined to yield 85 to 95% cell killing at 4 days post-infection. Antiviral activity and cytotoxicity were determined by XTT staining as described below.

**Anti-Herpes Simplex Type 1 Virus (HSV-1).** Vero cells, prepared as above, were resuspended at 5x10^3^ cells per mL and 100 μL added to each well to make a 70% confluent monolayer overnight. HSV-1_HF_ (VR-260) strain, obtained from ATCC, was diluted into the assay medium above such that the amount of virus added to each well in a volume of 100 μL was the amount determined to yield 85 to 95% cell killing at 6 days post-infection. Antiviral activity and cytotoxicity were determined by XTT staining as described below.

**Anti-Influenza Type A and B Virus (Flu A & B)**. MDCK cells (canine kidney cells, ATCC CCL-34) grown in DMEM supplemented with 10% FBS, 2 mM L-glutamine, 100 U/mL penicillin, 10 μg/mL streptomycin, 1 mM sodium pyruvate and 0.1 mM NEAA were resuspended at 1x10^4^ cells per mL and 100 μL added to each well. The microtiter plates were then incubated at 37°C in 5% CO_2_ overnight to allow for cell adherence. The influenza A/PR/8/34 (VR-95) and influenza B/Allen/45 (VR-102) was obtained from ATCC and a stock virus pool was produced in MDCK cells. Virus was diluted into the assay medium (DMEM supplemented with 0.5% BSA, 2 mM L-glutamine, 1 mM sodium pyruvate, 0.1 mM NEAA, 1 μg/mL TPCK-treated trypsin, 100 U/mL penicillin, 10 μg/mL streptomycin) such that the amount of virus added to each well in a volume of 100 μL was the amount determined to yield 85 to 95% cell killing at 4 days post-infection. Antiviral activity and cytotoxicity were determined by XTT staining as described below.

**Anti-Coronavirus (CoV).** MRC-5 cells (ATCC CCL-171) grown in the DMEM medium above were resuspended at 5x10^3^ cells per mL and 100 μL added to each well. The microtiter plates were then incubated at 37°C in 5% CO_2_ overnight to allow for cell adherence. The Coronavirus_229E_ strain (VR-740) was obtained from ATCC and grown in MRC-5 cells for the production of a stock virus pool. Virus was diluted into the assay medium (DMEM supplemented with 2% heat-inactivated FBS, 2 mM L-glutamine, 100 U/mL penicillin and 10 μg/mL streptomycin) such that the amount of virus added to each well in a volume of 100 μL was the amount determined to yield 85 to 95% cell killing at 6 days post-infection. Antiviral activity and cytotoxicity were determined by XTT staining as described below.

**Anti-Respiratory Syncytial Virus (RSV).** HEp2 cells (Human epithelial with HeLa, ATCC CCL-23) grown in DMEM supplemented with 10% FBS, 2 mM L-glutamine, 100 U/mL penicillin and10 μg/mL streptomycin were resuspended at 5x10^3^ cells per mL and 100 μL added to each well. The microtiter plates were then incubated at 37°C in 5% CO_2_ overnight to allow for cell adherence. The RSV_A2_ strain (VR-1540) was obtained from ATCC and grown in Hep2 cells for the production of a stock virus pool. Virus was diluted into the assay medium (DMEM supplemented with 2% heat-inactivated FBS, 2 mM L-glutamine, 1 mM sodium pyruvate, 0.1 mM NEAA, 100 U/mL penicillin, 10 μg/mL streptomycin) such that the amount of virus added to each well in a volume of 100 μL was the amount determined to yield 85 to 95% cell killing at 6 days post-infection. Antiviral activity and cytotoxicity were determined by XTT staining as described below.

**Anti-Human Rhinovirus (HRV).** H1-HeLa cells (ATCC CRL-1958) grown in the DMEM medium above were resuspended at 5x10^3^ cells per mL and 100 μL added to each well. The microtiter plates were then incubated at 37°C in 5% CO_2_ overnight to allow for cell adherence. The HRV26 strain 5560 (VR-501) was obtained from ATCC and grown in H1-HeLa cells for the production of a stock virus pool. Virus was diluted into the assay medium (DMEM supplemented with 2% heat-inactivated FBS, 2 mM L-glutamine, 100 U/mL penicillin, 10 μg/mL streptomycin) such that the amount of virus added to each well in a volume of 100 μL was the amount determined to yield 85 to 95% cell killing at 6 days post-infection. Antiviral activity and cytotoxicity were determined by XTT staining as described below.

**Anti-Adenovirus (AdV).** HeLa cells (ATCC CCL-2) grown in the DMEM medium above were resuspended at 5x10^3^ cells per mL and 100 μL added to each well. The microtiter plates were then incubated at 37°C in 5% CO_2_ overnight to allow for cell adherence. The Adenovirus_Tonsil99_ strain (VR-6) was obtained from ATCC and grown in HeLa cells for the production of a stock virus pool. Virus was diluted into the assay medium above such that the amount of virus added to each well in a volume of 100 μL was the amount determined to yield 85 to 95% cell killing at 6 days post-infection. Antiviral activity and cytotoxicity were determined by XTT staining as described below.

**Determination of antiviral activity and cytotoxicity.** Following incubation at 37^o^C in a 5% CO_2_ incubator, test plates were stained with the tetrazolium dye XTT (2,3-bis(2-methoxy-4-nitro-5-sulfophenyl)-5-[(phenylamino)carbonyl]-2H-tetrazolium hydroxide). XTT-tetrazolium was metabolized by the mitochondrial enzymes of metabolically active cells to a soluble formazan product, allowing rapid quantitative analysis of the inhibition of virus-induced or test article-induced cell killing by the antiviral test substances. XTT solution was prepared daily as a stock of 1 mg/mL in RPMI1640. Phenazine methosulfate (PMS) solution was prepared at 0.15 mg/mL in PBS and stored in the dark at -20^o^C. XTT/PMS stock was prepared immediately before use by adding 40 μL of PMS per ml of XTT solution. Fifty microliters of XTT/PMS was added to each well of the plate and the plate was reincubated for 4 hours at 37^o^C. Plates were sealed with adhesive plate sealers and shaken gently or inverted several times to mix the soluble formazan product and the plate was read spectrophotometrically at 450/650 nm with a Molecular Devices Vmax plate reader. The raw data was collected from Softmax Pro and imported into a Microsoft Excel XLfit4 spreadsheet for analysis using four parameter curve fit calculations to determine the percentage of cell viability and percentage of reduced viral CPE at each test concentration.

**Anti-HBV evaluation.** A volume of 100 µL of AD38 cells (gift from Dr. P. Furman) in RPMI 1640 medium with 10% FBS was added to all 96 wells of a microtiter plate at a density of 1x10^4^ cells per well and incubated at 37°C in 5% CO_2_ for 24 h. The test compound was serially diluted in the RPMI/FBS medium and added to the wells in triplicate (with six wells receiving medium alone as a virus only control). The plate was incubated for 6 days, with a change in medium (+/- compound) on Day 3. Supernatant (100 μL) was collected from each well for analysis of viral DNA by qPCR as described below and cytotoxicity was evaluated by XTT staining of the cell culture monolayer on the sixth day as described above.

**Quantitative PCR detection of HBV DNA.** Cell culture supernatant (10 μL) was diluted in qPCR dilution buffer (40 μg/mL sheared salmon sperm DNA) and boiled for 15 min. Quantitative real time PCR was performed in 386-well plates using an Applied Biosystems 7900HT Sequence Detection System and the supporting SDS 2.4 software. Samples (5 μL boiled DNA) and DNA standards were subjected to real time qPCR using Platinum Quantitative PCR SuperMix-UDG (Invitrogen) and specific DNA oligonucleotide primers (IDT, Coralville, ID) HBV-AD38-qF1 (5’-CCG TCT GTG CCT TCT CAT CTG-3’), HBV-AD38-qR1 (5’-AGT CCA AGA GTY CTC TTA TRY AAG ACC TT-3’), and HBV-AD38-qP1 (5’-FAM-CCG TGT GCA/ZEN/CTT CGC TTC ACC TCT GC-3’BHQ1) at a final concentration of 0.2 μM for each primer in a total reaction volume of 15 μL. The HBV DNA copy number in each sample was interpolated from the standard curve by the SDS 2.4 software and the data was imported into an Excel spreadsheet for analysis.
