# Supplementary material for: Preclinical evaluation of AT-527, a novel guanosine nucleotide prodrug with potent, pan-genotypic activity against hepatitis C virus
Source: PLoS One. 2020 Jan 8;15(1):e0227104. doi: 10.1371/journal.pone.0227104 (PMC6949113; doi:10.1371/journal.pone.0227104)
Supplement: S3 Protocol — (DOCX) [file pone.0227104.s017.docx]

**S3 Protocol.** **LC-MS/MS analysis of AT-511 and metabolites in cultured cells.** The supernatant extracts of each hepatocyte and Huh-7 cell incubation were separately analyzed by four different LC-MS/MS methods for quantitation of phosphoramidates, nucleosides, monophosphates, and triphosphates (methods 1, 2, 3 and 4, respectively), using synthetic reference standards of actually and potentially present compounds and using structurally related analogs as internal standards. Briefly, a Luna PFP (2), 5 µm, 100 x 4.6 mm column and an AB Sciex API-6500 mass spectrometer (ESI positive ion, MRM mode) were used for methods 1-3 and an XBridge Oligonucleotide, BEH C18, 2.5 µm, 50 x 2.1 mm column and a Thermo QE Plus mass spectrometer (electrospray, negative ion mode) were used for method 4. For methods 1 and 2, mobile phases A (10 mM ammonium acetate in water) and B (acetonitrile:methanol, 1:1, v/v) were used to elute samples (5 µL) at 1 mL/min with 15% B for 1.5 min followed by a linear gradient to 75% B over 1 min (method 1) or with 54% B for 1.3 min followed by a linear gradient to 90% B over 1.2 min. Method 3 was the same except mobile phase A was 0.3% acetic acid in water, pH 8 and samples were eluted with 3% B for 3 min followed by a linear gradient to 55% B over 1.2 min. For method 4, mobile phase A (3 mM ammonium formate with 10 mM dimethylhexylamine (DMHA) in water) and mobile phase B (3 mM ammonium formate with 10 mM DMHA in acetonitrile:water, 1:1, v/v) were used to elute samples at 0.4 mL/min with 0% B for 1.5 min followed by a linear gradient to 100% B over 2 min and ending with an isocratic elution for 0.5 min at 100% B.
